# Decreased Efficacy of Doxorubicin Corresponds With Modifications in Lipid Metabolism Markers and Fatty Acid Profiles in Breast Tumors From Obese vs. Lean Mice

**DOI:** 10.3389/fonc.2020.00306

**Published:** 2020-03-17

**Authors:** Ilze Mentoor, Theo Nell, Zaakiyah Emjedi, Paul J. van Jaarsveld, Louis de Jager, Anna-Mart Engelbrecht

**Affiliations:** ^1^Department of Physiological Sciences, Faculty of Natural Sciences, University of Stellenbosch, Stellenbosch, South Africa; ^2^Non-Communicable Diseases Research Unit, South African Medical Research Council, Cape Town, South Africa; ^3^Division of Medical Physiology, Faculty of Medicine and Health Sciences, Stellenbosch University, Tygerberg, South Africa; ^4^Division of Anatomical Pathology, Faculty of Medicine and Health Sciences, Stellenbosch University, Tygerberg, South Africa

**Keywords:** obesity, breast cancer, adipose tissue, fatty acids, treatment efficacy

## Abstract

Breast cancer cells modulate lipid and fatty acid metabolism to sustain proliferation. The role of adipocytes in cancer treatment efficacy remains, however, to be fully elucidated. We investigated whether diet-induced obesity (DIO) affects the efficacy of doxorubicin treatment in a breast tumor-bearing mouse model. Female C57BL6 mice were fed a high fat or low fat diet for the full duration of the study (12 weeks). After 8 weeks, mice were inoculated with E0771 triple-negative breast cancer cells in the fourth mammary gland to develop breast tumor allographs. Tumor-bearing mice received either vehicle (Hank's balanced salt solution) or doxorubicin (chemotherapy). Plasma inflammatory markers, tumor, and mammary adipose tissue fatty acid composition, as well as protein expression of lipid metabolism markers were determined. The high fat diet (HFD) attenuated the treatment efficacy of doxorubicin. Both leptin and resistin concentrations were significantly increased in the HFD group treated with doxorubicin. Suppressed lipogenesis (decreased stearoyl CoA-desaturase-1) and lipolysis (decreased hormone-sensitive lipase) were observed in mammary adipose tissue of the DIO animals, whereas increased expression was observed in the tumor tissue of doxorubicin treated HFD mice. Obesogenic conditions induced altered tissue fatty acid (FA) compositions, which reduced doxorubicin's treatment efficacy. In mammary adipose tissue breast cancer cells suppressed the storage of FAs, thereby increasing the availability of free FAs and favored inflammation under obesogenic conditions.

## Introduction

The incidence of lifestyle associated conditions including obesity is a rising epidemic (1), this is especially alarming since breast cancer remains a major health risk for women globally (2). Obesity is identified as a casual factor in both the development as well as the progression of breast carcinogenesis (3, 4), and is characterized by rapid adipose tissue remodeling (hypertrophy and hyperplasia) (5), increased synthesis of several adipokines such as leptin, resistin, tumor necrosis factor-alpha (TNF-α), interleukin (IL)-1β, IL-6, macrophage chemoattractant protein-1 (MCP-1), and immune cell infiltration, all of which lead to a state of sustained low-grade inflammation. Mammary adipose tissue serves as a exogenous source of energy metabolites which favors the proliferation demand of breast cells in the tumor microenvironment (6, 7). On the other hand, breast cancer cells can also modulate lipid metabolism by altering both *de novo* fatty acid (FA) synthesis as well as the catabolic break down of triacylglycerol's (TAGs) a process known as lipolysis. This subsequently results in the release of free fatty acids (FFAs) which become available metabolic substrates for the benefit of breast cancer cell survival, either by storage in the form of lipid droplets, membrane lipids or energy production *via* β-oxidation supplying energy to these proliferating breast cancer cells (6, 8).

The role of FAs in cancer progression and treatment resistance implicates various physiological functions of FAs in relation to both dietary intake and *de novo* synthesized FAs. It is proposed to be achieved by *(i) alterations in cell membrane composition, (ii) the biosynthesis of lipid-signaling molecules, and (iii) its role in metabolic reprogramming as an energy source* [reviewed in (9, 10)]. Both SFAs and MUFAs are implicated in alterations within cancer cell membrane composition known as *membrane lipid saturation*. These FA classes are more resistant to lipid peroxidation, which in turn protects cancerous cells against oxidative stress induced by therapies (11, 12). The role of omega-6 (n-6) PUFAs in breast cancer development, progression as well as treatment resistance, includes n-6 PUFAs exhibiting pro-inflammatory effects mediated by lipid-derived bioactive mediators i.e., eicosanoids, prostaglandins and leukotrienes (13, 14). These lipid-derived bioactive mediators upregulate signaling pathways that are involved in inflammation, which exacerbate angiogenesis, cell-proliferation and inflammation (15), to contribute to an ideal microenvironment favoring mammary carcinogenesis.

Recently, findings from cell culture and animal models identified obesity as a main contributing factor in the underlying pathophysiology implicated in the development of breast cancer chemotherapeutic drug resistance (16, 17). Patients suffering from obesity and breast cancer presented with poor clinical outcomes when treated with first line adjuvant regimens such as doxorubicin (18, 19). Despite doxorubicin's high efficacy in killing cancer cells, its' clinical efficacy is hindered by the development of various cellular toxicities which contributes to the development of chemotherapeutic drug resistance (20). Doxorubicin treatment is also associated with cellular toxicities in adipose tissue (primary storage site for FAs) which in turn leads to dysfunctional lipid/FA storage (21, 22). Therefore, FA tissue composition may also be significantly altered by chemotherapeutic agents.

A lack of evidence highlighting the role of FAs in breast cancer treatment efficacy, as well as an incomplete understanding of cellular mechanisms whereby obesity affects chemotherapy outcomes, necessitates further investigation. We therefore aimed to determine whether diet-induced obesity (DIO) affects the efficacy of doxorubicin treatment in a breast tumor-bearing mouse model and to explore possible mechanisms of action.

## Methods

Female C57BL6 mice were fed a low fat diet (LFD) or a high fat diet (HFD) for 12 weeks. After developing the DIO phenotype, syngeneic breast tumors were induced, followed by respective treatments.

### Animals and Handling

Animal handling and interventions were carried out under the supervision of a registered small animal handling expert at the Stellenbosch University. Ethical clearance was obtained from Stellenbosch University animal research committee (SU-ACUM13-00015). All protocols strictly adhered to the standard care guidelines of laboratory animals implemented at Stellenbosch University and according to the South African National Standards 10386:2008 for the use of animals in research and teaching.

Three-week-old female C57BL6 mice (*n* = 40) were maintained in the animal research facility at the University of Stellenbosch in static micro-isolation sterilized cages (*n* = 5 per cage) with filtered air. The mice were provided with chow and water *ad libitum* in a regular 12:12 h light-dark cycle. All animals were acclimated for 1 week followed by the assignment to either HFD or LFD groups. The general welfare of all animals were monitored daily.

### Diet Regimens

**Figure 1:**
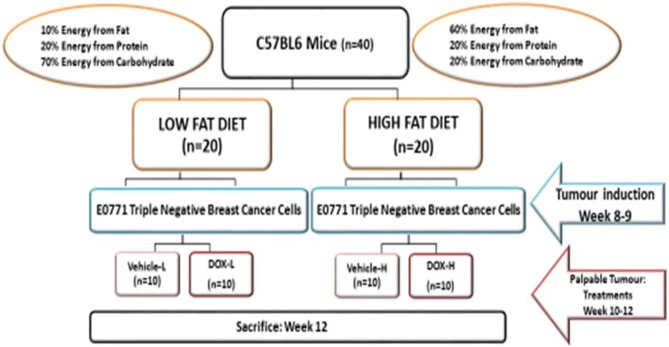
Representative summary of the *in vivo* model and respective experimental groups.

A HFD was used to induce obesity since reported evidence showed that genetic models of obesity (i.e., *ob/ob, db/db*, and *leptin*/ *leptin* receptor-deficient mice) demonstrated resistance in developing mammary cancer (23). C57BL6 mice are particularly sensitive to DIO (24). Forty mice (*n* = 40) were randomly assigned into two equal groups (*n* = 20) and allocated one of two respective diets for 12 weeks (Figure 1). The energy content of the HFD (D12492, Research diet Inc., New Jersey, USA) consisted of 60% energy from fat, 20% energy from protein, and 20% energy from carbohydrates, compared to the LFD (D12450J, Research diet Inc., New Jersey, USA), containing 10% energy from fat, 20% energy from protein, and 70% energy from carbohydrates (Table 1). The dietary FA composition of the respective diets is summarized in Supplementary Table 1. Body weight was monitored weekly over the study period and the DIO phenotype was confirmed after 8 weeks followed by tumor induction.

**Table 1:** Dietary composition of low fat diet and high fat diet.

	**Low Fat Diet (LFD)**		**High Fat Diet (HFD)**	
	**Research diet D12450J**		**Research diet D12492**	
	**gram%**	**kcal%**	**gram%**	**kcal%**
Protein	19.2	20	26.2	20
Carbohydrates	67.3	70	26.3	20
Fat	4.3	10	34.9	60
Total		100		100
Kcal/gm	3.85		5.24	
**INGREDIENTS**	**gram**	**kcal**	**gram**	**kcal**
Casein, 30 Mesh 200	200	800	200	800
L-Cystine	3	12	3	12
Corn starch	506.2	2024.8	0	0
Maltodextrin 10	125	500	125	500
Sucrose	68.8	275.2	68.8	275.2
Cellulose BW200	50	0	50	0
Soybean oil	25	225	25	225
Lard	20	180	245	2,205
Mineral mix S10026	10	0	10	0
Dicalcium phosphate	13	0	13	0
Calcium carbonate	5.5	0	5.5	0
Potassium citrate, 1 H_2_O	6.5	0	6.5	0
Vitamin mix V10001	10	40	10	40
Choline bitartrate	2	0	2	0
FD&C yellow dye #5	0.04	0		
FD&C blue dye #1	0.01	0	0.05	0
TOTAL	1055.1	4,057	773.9	4057.0
Cholesterol (mg)/4057 kcal	**–**	54.4	**–**	216.4
Cholesterol (mg)/kg	**–**	51.6	**–**	279.6

*As per manufacturer product data sheet (Research diet Inc., New Jersey, USA)*.

### Tumor Induction

#### Cell Culture

An aggressive triple-negative breast cancer cell line with metastatic capabilities (E0771) that originated from a tumor after a spontaneous mutation in a C57BL6 mouse, was used in this *in vivo* model. The cells were cultured in T75 flasks (75 cm^2^, SPL Life Sciences, Pocheon-si, South Korea) with Dulbecco's Modified Eagle's medium (DMEM, Gibco®, ThermoFisher Scientific, Massachusetts, United States) under standard incubation conditions (37°C and 5% CO_2_ humidity), supplemented with 10% fetal bovine serum (FBS, Capricorn Scientific, Germany) and 1% Penicillin Streptomycin (PenStrep Gibco, ThermoFisher Scientific, Massachusetts, United States). Growth media was replaced every 2 day. Regular sub-culturing was performed once cultures reached 70–80% confluency.

#### Inoculation of Tumors

E0771 cells were prepared for each mouse. The mice were anesthetized under 3% (v/v) isoflurane (Isofor, Safeline Pharmaceuticals, Johannesburg, South Africa) in an anesthetic chamber. Mice were inoculated subcutaneously (using a 23-gauge needle syringe) in the fourth left mammary fat pad with 1.2 × 10^5^ E0771 triple-negative breast cancer cells suspended in Hanks Balanced Salt Solution (HBSS) (Sigma Chemical Co., St Louis, MO, USA) as illustrated in Figure 1.

#### Drug Administration

Once tumors became palpable (200–300 mm^2^), LFD and HFD mice were randomly assigned to the respective treatment groups (Figure 1). The treatment groups included: (1) vehicle control (isovolumetric intra-peritoneal injection of HBSS), and (2) doxorubicin treatment (D5794, LKT® laboratories, Minnesota, USA). Mice were restrained and treated with three dosages of 4 mg/kg doxorubicin (cumulative dosage of 12 mg/kg) *via* intraperitoneal injection. The dosage of 12 mg/kg doxorubicin is equivalent to 36 mg/m^2^ in humans which is within the clinically relevant dosage range of doxorubicin treatment (15–90 mg/m^2^) (25).

The experimental groups were assigned as follows: (i) tumor vehicle-LFD (vehicle-L), (ii) tumor vehicle-HFD (vehicle-H), (iii) tumor doxorubicin-LFD (Dox-L), and (iv) tumor doxorubicin-HFD (Dox-H). Humane endpoints were implemented when tumor growth influenced the general welfare or restricted mobility of the mice, or when the mice began to bite their tumors and exhibit changes in posture and facial expression, as determined by the grimace scale. The final sample size per experimental group were as follows: vehicle-L (*n* = 8), vehicle-H (*n* = 9), Dox-L (*n* = 10), and Dox-H (*n* = 9).

#### Measurements, Blood Collection, and Tumor- and Fat Tissue Excision

Every second day, animals were weighed and tumor location and volume were recorded. The absolute body weight was calculated after subtracting tumor weight. Tumor growth was measured using a Harpenden caliper (in mm) to determine tumor volume using the following equation:


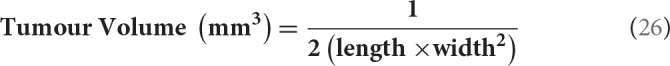


Animals were euthanised 72 h after the last scheduled doxorubicin administration. Mice were anesthetized under 3% isoflurane and sacrificed by cervical dislocation after a deep sleep was confirmed by the absence of pedal reflex. Whole blood was immediately collected into pediatric EDTA tubes (Lasec, Cape Town, South Africa) from the thoracic cavity. Collected blood samples were placed on ice and centrifuged (1,000 RCF (g), 10 min), to collect and aliquot plasma which was stored at −80°C for subsequent analysis. Mammary adipose tissue was collected from the third and fourth quadrant of the mice and tumor tissue were dissected, weighed, snap-frozen with liquid nitrogen and stored at −80°C or stored in formalin at room temperature for immunohistochemistry analysis.

#### Blood Analysis

Plasma samples were used to quantify TNF-α, IL-6, IL-10, leptin (PPX-04-MXCE327, Thermo Fisher Scientific, United States), IL-1β and vascular endothelial growth factor (VEGF-A) (PPX-02-MXFVKXT, Thermo Fisher Scientific, United States) using a custom ProcartaPlex panel and matched mouse Luminex kits. A Milliplex mouse adipokine magnetic bead panel MAP kit was used to quantify MCP-1, insulin, total plasminogen activator inhibitor-1 (PAI-1) and resistin (MADKMAG-71K, Burlington, Massachusetts, United States). All analyses were performed according to the manufacturers' protocols and specifications. Analytes were measured simultaneously using a MAGPIX system plate reader (APX1042, Bio-Rad, California, United States) and data (expressed in pg/ml) was processed on Bioplex Software 6.1 (Bio-Rad, California, United States).

### Determination of Tissue Fatty Acid Profiles

For tumor tissue, FA composition of the total phospholipid (TPL) and the FFA fractions were determined, whereas for the mammary adipose tissue, the total lipid FA composition was determined. Frozen tumor tissue and mammary adipose tissue were allowed to thaw at room temperature. Approximately 100 mg of tumor tissue and 30 mg of adipose tissue were weighed for lipid extraction using chloroform:methanol (C:M; 2:1; v:v; Sigma-Aldrich, St. Louis, Missouri, United States) according to a method adapted from Folch et al. (27) as previously described by Hon et al. (28). The extraction solvent contained 0.01% butylated hydroxytoluene (Sigma-Aldrich, St. Louis, Missouri, United States), acting as an antioxidant.

Briefly, lipids of tumor tissue were extracted with 9 mL of C:M (2:1; v:v) by homogenisation for 1 min using a Polytron® PT-MR 3100D homogeniser (Kinematica, Luzern, Switzerland). The homogenate was filtered through a sintered glass funnel with the filter pad lined with a glass microfiber filter disk (GF/A, Whatman, England) into a round bottom flask. The Polytron® shaft was rinsed with another 7 mL of the extracting solvent and filtered, collecting the rinse into a round bottom flask. The microfiber filter disk containing the homogenized tissue was removed and placed into an extraction tube and extracted again with 10 mL C:M (2:1; v:v) by 20-min shaking and a filtering step (repeated twice). The combined extraction phases containing the lipids were concentrated to dryness through rotary evaporation in a 37°C water bath (BÜCHI Labortechnik, Postfach, Switzerland). Lipids were transferred from the round bottom flask to a 12 mL glass tube with screw cap using 5 × 2 mL chloroform:methanol:saline (CMS; 86:14:1; v:v:v; Sigma-Aldrich) transfer volumes. Saline saturated with CMS (1 mL) was added, mixed and centrifuged, and the top saline layer was completely removed in order to concentrate the bottom phase to dryness under nitrogen gas flow in a 37°C water bath.

Neutral lipids were separated from the TPL fraction using thin-layer chromatography (TLC) silica gel 60 plates (10 × 10 cm; No. 1.05626.0001; Merck, Darmstadt, Germany) and eluted with the solvent system petroleum ether (B&M Scientific, Cape Town, South Africa):diethyl ether (Merck):acetic acid (Merck) (90:30:1; v:v:v). The lipid bands containing the TPL and FFA fractions were demarcated by visualization under long-wave UV light after plates were sprayed with C:M (1:1; v:v) containing 2,5-bis-(5′-tert-butylbenzoxazolyl-[2′]) thiophene (10 mg/100 mL; Sigma-Aldrich). These lipid bands were scraped off the plates into glass tubes with screw caps. The lipids were trans-esterified through trans-methylation with 2 mL methanol:sulphuric acid (H2SO4; BDH Chemicals, Poole, England) (95:5; v:v) at 70°C for 2 h to yield FA methyl esters (FAMEs). After cooling, the FAMEs were extracted with distilled water (1 mL) and n-hexane (3 mL) (Sigma-Aldrich). The upper hexane layer containing the FAMEs was collected and evaporated to dryness for subsequent gas-liquid chromatography (GLC) analysis.

Total lipids were extracted from mammary adipose tissue with 9 mL C:M (2:1; v:v) by shaking for 20 min with a mechanical shaker. Subsequently, 1.8 mL saline saturated with CMS was added, mixed and centrifuged at 60 RCF (g) for 10 min at 4°C. The bottom phase was collected and transferred to a 12 mL glass tube with a screw cap and the lipid extract evaporated to dryness under nitrogen gas flow using a 37°C water bath. The dried lipids were re-dissolved in 3 mL C:M (2:1; v:v) of which a 50 μL aliquot was transferred to a clean 12 mL glass tube and the lipid aliquot was evaporated to dryness as described before. These lipids were trans-methylated with 2 mL methanol:sulphuric acid (70°C for 2 h) with subsequent sample FAME isolation for GLC analysis as described above.

All FAMEs were re-dissolved in n-hexane and analyzed (sample injection volume 1 μl) by GLC on a Finnigan Focus Gas Chromatograph (Thermo Electron Corporation, Austin, TX, USA) equipped with a flame-ionization detector and a 30 m capillary column of 0.32 mm internal diameter (BPX70 0.25 μm; SGE International, Ringwood, Victoria, Australia). Gas flow rates were: N2 (make up gas), 25 mL/min; synthetic air, 250 mL/min; and H2 (carrier gas), 25 mL/min, with a 20:1 split ratio. Oven temperature programming was linear at 4.5°C/min, initial temperature 140°C (hold-time 1 min), final temperature 220°C (hold-time 5 min), injector temperature 220°C, and detector temperature 250°C [as previously described (29)].

All sample FAMEs were subsequently identified by analyzing and comparing sample retention times with those a known standard FAME mixture (27 FAMEs, NuChek Prep, Elysian, MN, USA). Relative percentages of each individual FAME was calculated by determining the area count of a specific FAME as a percentage of the total area count of all FAMEs identified in the sample. Estimated desaturase indexes were estimated by product to precursor FA ratios which included stearoyl CoA-desaturase-1 (SCD1)-16 calculated as the ratio of palmitoleic acid (PTA) to palmitic acid (PA) and SCD1-18 calculated as the ratio of oleic acid (OA) to stearic acid (SA) (30, 31).

### Protein Analysis and Western Blot Analysis

Mammary adipose tissue and tumor tissue samples were placed on ice and allowed to thaw at 4°C. Total protein extraction was performed where samples were suspended in 300 μl of cold modified radio-immunoprecipitation (RIPA) assay buffer containing protease and phosphatase inhibitors (2.5 mM Tris-HCL, 0.1 mM phenylmethylsulfonyl fluoride, 10 mg/ml leupeptin, 1 mM EDTA, 1 mM benzamidine, 50 mM sodium fluoride, 1 mM dithiothreitol, 4 mg/ml soybean trypsin inhibitor, 0.1% sodium dodecyl sulfate (SDS), 0.5% sodium deoxycholate, and 1% NP-40, pH 7.4). Samples were homogenized on ice under sterile conditions to prevent protein cross-contamination. Next, all samples were centrifuged (35,000 RCF (g), 60 min, 4°C), to yield distinct layers. The supernatant layer was removed using a sterile 23-gauge needle and syringe and transferred into sterile Eppendorf tubes, followed by another centrifugation step (35,000 RCF (g), 30 min, 4°C). The process of removing the supernatant was repeated and samples were run through Amicon® Ultra 0.5 mL filters (Merck, Darmstadt, Germany) for protein purification and concentration and stored at −80°C, until protein quantification using a Direct Detect® infrared spectrometer (DDHW00010-WW, Merck). This was followed by preparation of protein aliquots containing 20–50 μg protein diluted with Laemmli sample buffer and boiled for 5 min (to denature proteins) before being loaded into 4–15% polyacrylamide fast cast gels (mini-PROTEAN® TGX™ Gels, Bio-Rad) for separation by sodium dodecyl sulfate polyacrylamide gel electrophoresis (SDS-PAGE). Gels were run at 100 V (constant) and 400 mA for approximately 60 min (Power Pac 300, BioRad). The electro-transfer of proteins from the gel to prepared polyvinylidene fluoride (PVDF) membranes was achieved using a semi-dry electro-transfer system (TransBlot® Turbo™ v1.02, BioRad) for 30 min at 25 V and 1.0 A. Transfer efficiency was evaluated using the stain-free blot protocol provided on a Chemi-Doc™ MP (BioRad) system. Subsequently, all membranes were washed with 0.1% Tris Buffered Saline-Tween 20 (TBS-T) and blocked for 60 min in 5% (w/v) non-fat milk and TBS-T at room temperature to prevent non-specific binding. The PVDF membranes were then incubated overnight in primary antibody solutions (1:1,000, diluted in 5% w/v BSA, 1X TBS-T, refer to Supplementary Table 2) at 4°C. The following day, membranes were washed three times for 5 min each with TBS-T, prior to incubation with an anti-rabbit IgG horseradish peroxidase conjugated secondary antibody (1:10,000) (Cell Signaling Technologies, Massachusetts, United States), for 60 min at room temperature. A wash step followed, using TBS-T (five times for 5 min each), before specific bands were visualized and detected using the enhanced chemiluminescence (ECL) western blotting substrate detection kit (Pierce®, Thermo Scientific) and ImageLab 4.0 software on a Chemi-Doc™ MP (BioRad) imaging system. Protein quantification of samples were normalized to total protein signal in each lane present on the same membrane after blotting (ImageLab 4.0 software, Biorad USA), as determined by the Stain-Free™ (ImageLab 4.0 software, Biorad USA) properties of the blot and is expressed as a percentage of the control.

### Haematoxylin and Eosin Stained Tumor Tissue

Sectioning, deparaffinization and rehydration of tumor tissue samples was performed as previously described (32). Tumor tissue samples were stained for histological changes using haemotoxylin and eosin (H&E) staining. Staining was achieved by using an automated tissue stainer (Leica Biosystems, ST4020), during which section slides where dipped into haemotoxylin. This was followed by various subsequent 2-min dipping steps in distilled water, scott's tap water, distilled water, eosin and distilled water followed by coverslips being mounted using DPX mounting media.

### Statistical Analysis

Statistical analyses were performed using Statistica version 13.3 (TIBCO Software, California, United States). Normality was assessed using the Shapiro-Wilk test and results were reported as mean ± standard error of the mean (SEM). To describe differences between two groups, *t*-tests were used, and to describe differences between the three/more groups two- or three-way ANOVA were used, followed by the Fisher's LSD *post-hoc* test. Pearson's correlations were used on selected parameters in each group and 2D scatter plots were drawn up in GraphPad Prism version 7 (GraphPad Software, San Diego, United States). Statistical significance was accepted at *p* < 0.05.

## Results

### A High Fat Diet Increased Body Weight and Mammary Adipose Tissue Weight

#### Body Weight and Food Consumption

**Figure 2:**
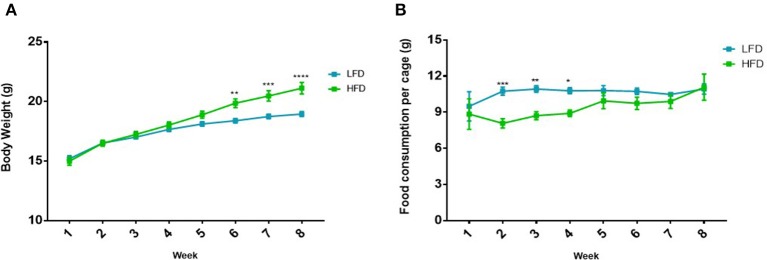
Difference in **(A)** body weight and **(B)** food consumption in mice (*n* = 5 per cage) on the LFD and HFD for 8 weeks. Results are presented as mean ± SEM (*n* = 20 per group). *T*-tests were used for comparison between the LFD and the HFD mice for all weeks and *p* < 0.05 was considered as statistically significant. *p < 0.05, **p < 0.01, ***p < 0.001 and ****p < 0.0001.

During the DIO period, mice that were fed the HFD showed significantly higher body weights at week 6 (*p* < 0.01), week 7 (*p* < 0.001) and week 8 (*p* < 0.0001), compared to the LFD group (Figure 2A), therefore DIO was established after 8 weeks. It was also observed that mice fed a HFD showed significantly lower food consumption per cage at week 2 (*p* < 0.001), 3 (*p* < 0.01) and 4 (*p* < 0.05) compared to the LFD group (Figure 2B).

**Figure 3:**
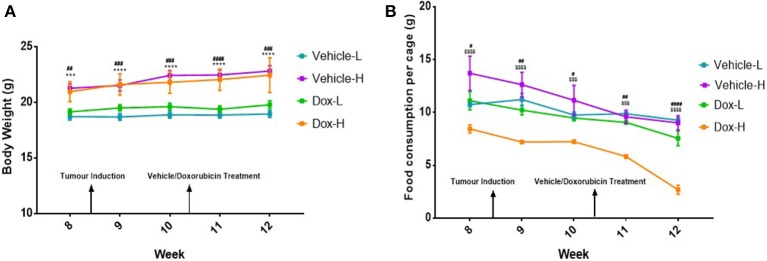
**(A)** Mean body weight, and **(B)** food consumption of mice (*n* = 5 per cage) receiving vehicle-or doxorubicin treatment while on the LFD control compared to the HFD. Results are presented as mean ± SEM (*n* = 10 per group). Three-way ANOVA with Fisher's LSD *post hoc* correction was applied and *p* < 0.05 was considered as statistically significant. ***p < 0.001 and ****p < 0.0001. ^#^*p* < 0.05, ^##^*p* < 0.01, ^####^*p* < 0.0001, ^$$$^*p* < 0.001 and ^$$$$^*p* < 0.0001. *Vehicle-L vs. Vehicle-H, ^#^Dox-L vs. Dox-H, and ^$^Vehicle-H vs. Dox-H.

Following tumor induction, mice in the vehicle-H group showed significantly higher body weight compared to vehicle-L mice during week 8–12 (all *p* < 0.001) (Figure 3A). A similar and statistically significant observation was made for body weight of mice in the Dox-H group, when compared to Dox-L mice at week 8–12 (all *p* < 0.01) (Figure 3A). The Dox-H mice also showed significantly lower food consumption compared to the vehicle-H mice at week 8 (*p* < 0.0001), 9 (*p* < 0.0001), 10 (*p* < 0.001), 11 (*p* < 0.001), and 12 (*p* < 0.0001) (Figure 3B). Lastly, the Dox-H mice revealed significantly lower food consumption than the Dox-L mice at week 8 (*p* < 0.05), 9 (*p* < 0.01), 10 (*p* < 0.05), 11 (*p* < 0.01), and 12 (*p* < 0.0001) (Figure 3B).

### Mammary Adipose- and Tumor Tissue Weight

**Figure 4:**
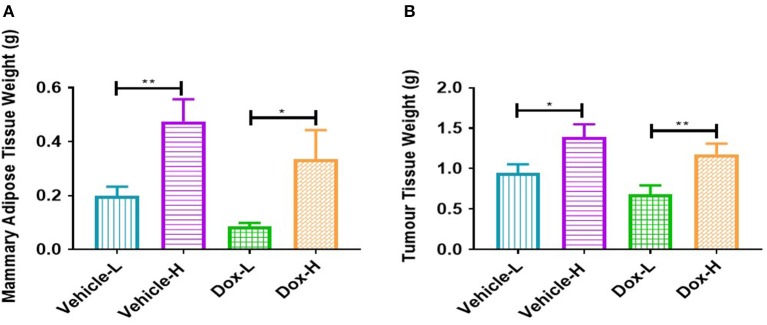
Differences in **(A)** mammary adipose tissue weight, and **(B)** tumor weight of vehicle- and doxorubicin-treated groups on LFD control compared to HFD. Results are presented as mean ± SEM (*n* = 9–10 per group). Two-way ANOVA with Fisher's LSD *post hoc* correction were applied. *p < 0.05, **p < 0.01.

The vehicle-H mice showed significantly higher mammary adipose tissue weight (*p* < 0.01) and tumor weight (*p* < 0.05) in comparison to the vehicle-L mice (Figures 4A,B). Mice in the Dox-H group presented with significantly higher mammary adipose tissue weight (*p* < 0.05) as well as tumor weight (*p* < 0.01) compared to Dox-L mice (Figures 4A,B).

### Diet-Induced Obesity Decreased Doxorubicin Treatment Efficacy in Breast Tumors

**Figure 5:**
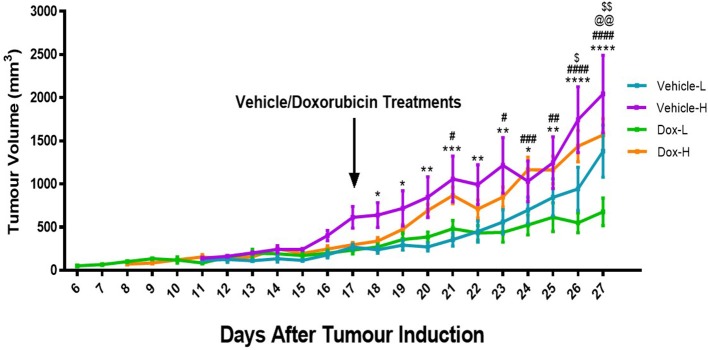
Differences in tumor volume for the vehicle control and doxorubicin treatment groups on LFD and HFD. Results are presented as mean ± SEM (*n* = 10 per group). Three-way ANOVA with Fisher's LSD *post hoc* correction was applied and *p* < 0.05 was considered as statistically significant. *p < 0.05, **p < 0.01, ***p < 0.001, and ****p < 0.0001. ^#^*p* < 0.05, ^##^*p* < 0.01, ^###^*p* < 0.001, and ^####^*p* < 0.0001, ^$^*p* < 0.05. ^*^Vehicle-L vs. Vehicle-H, ^#^Dox-L vs. Dox-H, ^@^Vehicle-L vs. Dox-L, ^$^Vehicle-H vs. Dox-H. ^@@^*p* < 0.01, ^$$^*p* < 0.01.

Mice in the vehicle-H group showed significantly higher tumor volume compared to corresponding vehicle-L mice at day 18 (*p* < 0.05), 19 (*p* < 0.05), 20 (*p* < 0.01), 21 (*p* < 0.001), 22 (*p* < 0.01), 23 (*p* < 0.01), 24 (*p* < 0.05), 25 (*p* < 0.01), 26 (*p* < 0.0001), and 27 (*p* < 0.0001), as illustrated in Figure 5.

Similarly, mice in the Dox-H group showed significantly higher tumor volume compared to corresponding mice from the Dox-L group, at day 21 (*p* < 0.05), 23 (*p* < 0.05), 24 (*p* < 0.001), 25 (*p* < 0.01), 26 (*p* < 0.0001), and 27 (*p* < 0.0001) (Figure 5). Dox-L mice also had significantly lower tumor volumes at day 27 compared to vehicle-L mice (*p* < 0.01), and Dox-H mice yielded significantly lower tumor volume at day 26 (*p* < 0.05) and 27 (*p* < 0.01) compared to the vehicle-H mice (Figure 5).

### Diet-Induced Obesity Induced Systemic Inflammation and Local Inflammatory Signaling in Mammary Adipose Tissue of Obese Mice Treated With Doxorubicin

**Figure 6:**
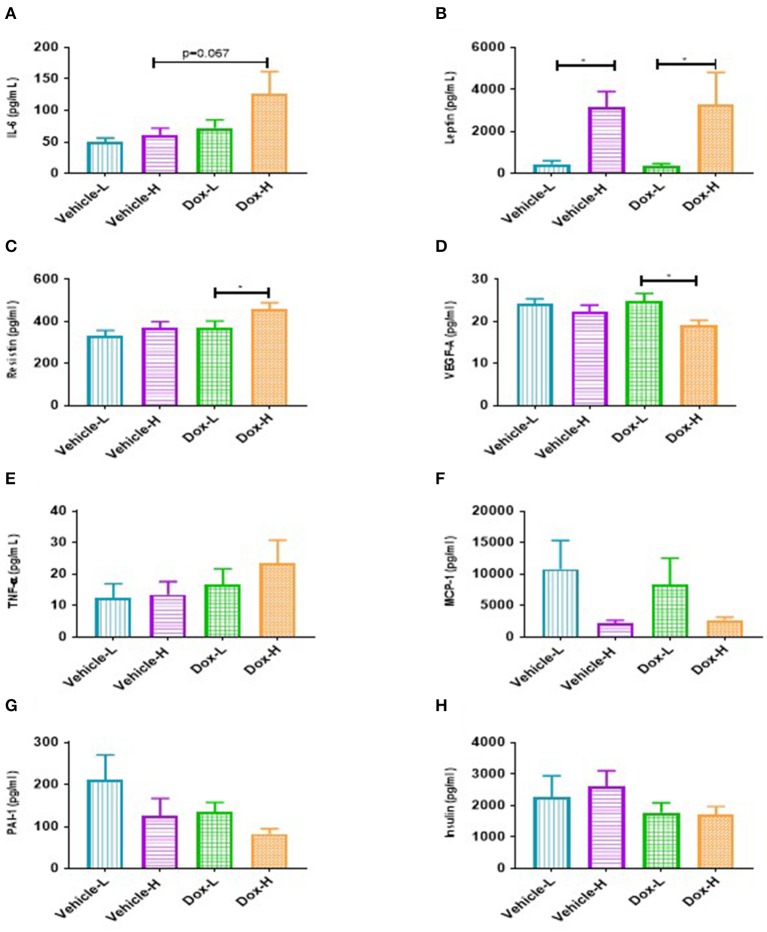
Mean inflammatory marker concentrations for vehicle control and doxorubicin treatment groups on LFD and HFD. **(A)**, IL-6 **(B)** Leptin, **(C)** Resistin, **(D)** VEGF-A, **(E)** TNF-α, **(F)** MCP-1, **(G)** PAI-1, and **(H)** Insulin. Results are presented as mean ± SEM (*n* = 6–9). Two-way ANOVA with Fisher's LSD *post hoc* correction was employed and *p* < 0.05 was considered as statistically significant. *p < 0.05.

A trend toward significance was observed for IL-6 in Dox-H mice compared to vehicle-H mice (*p* = 0.067, Figure 6A). Leptin levels were significantly higher in vehicle-H compared to vehicle-L mice (*p* < 0.05, Figure 6B) and Dox-H compared to Dox-L mice (*p* < 0.05, Figure 6B), respectively. Mice in the Dox-H group showed significantly higher resistin (*p* < 0.05, Figure 6C) and decreased VEGF-A levels (*p* < 0.05, Figure 6D) compared to Dox-L mice. No significant differences were reported for TNF-α (Figure 6E), MCP-1 (Figure 6F), PAI-1 (Figure 6G), and insulin (Figure 6H) between any of the respective experimental groups. Interleukin-10 and IL-1β were undetectable within all samples of all the experimental groups.

Pearson's correlation analysis revealed some positive correlations between leptin and mammary adipose tissue weight (Supplementary Figure 1). Significant strong positive correlations were only observed for the doxorubicin treatment groups (Dox-L, *r* = 0.78, *p* < 0.01, and Dox-H, *r* = 0.92, *p* = 0.001; Supplementary Figures 1C,D).

**Figure 7:**
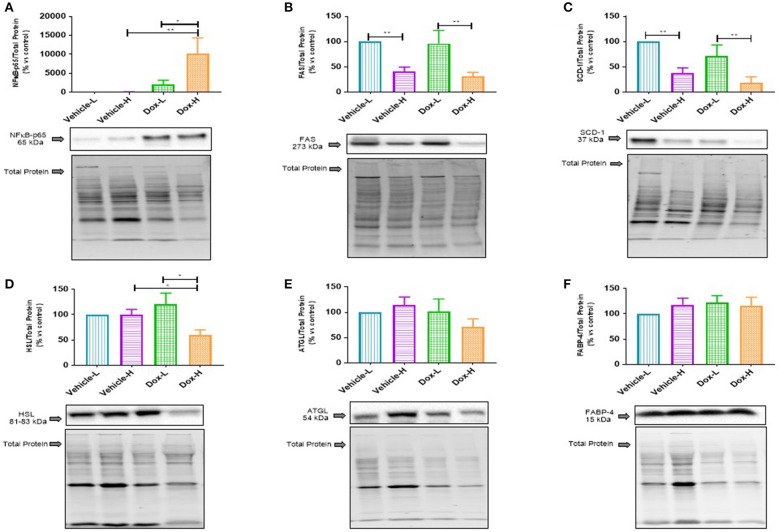
Western blot analysis of lipid metabolism marker protein expression in mammary adipose tissue of vehicle control and doxorubicin treatment groups on LFD and HFD; **(A)** NFκB-p65, **(B)** FAS, **(C)** SCD-1, **(D)** HSL, **(E)** ATGL, and **(F)** FABP4. Results are presented as mean ± SEM (*n* = 6–8). Two-way ANOVA with Fisher's LSD *post hoc* correction was employed and *p* < 0.05 was considered as statistically significant. *p < 0.05, **p < 0.01.

Lastly, Dox-H mice showed significantly higher protein expression of nuclear factor kappa B (NFκB-p65) compared to both Vehicle-H (*p* < 0.01) and Dox-L (*p* < 0.05) mice, respectively (Figure 7A).

### Diet-Induced Obesity and Doxorubicin Treatment Suppressed *De novo* Lipogenesis and Lipolysis in Mammary Adipose Tissue

Fatty acid synthase (FAS) and sterol CoA-desaturase-1 (SCD-1) were found to be significantly decreased in the vehicle-H mice, compared to vehicle-L mice (FAS, *p* < 0.01 and SCD-1, *p* < 0.01 Figures 7B,C) and Dox-H mice compared to Dox-L mice (FAS, *p* < 0.01, and SCD-1, *p* < 0.01 Figures 7B,C), respectively. Moreover, hormone-sensitive lipase (HSL) was significantly decreased in Dox-H compared to both vehicle-H (*p* < 0.05, Figure 7D) and Dox-L mice (*p* < 0.05, Figure 7D). No significant differences were observed for adipose triglyceride lipase (ATGL) (Figure 7E) and fatty acid binding protein 4 (FABP4) (Figure 7F), between any of the respective experimental groups (*p* > 0.05).

### Diet-Induced Obesity Increased *De novo* Lipogenesis and Lipolysis in Breast Tumors Treated With Doxorubicin

**Figure 8:**
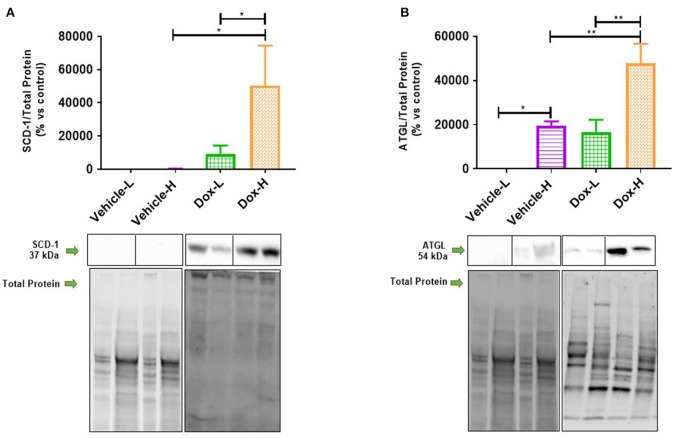
Western blot analysis of lipid metabolism protein expression in tumor tissue of vehicle control and doxorubicin treatment groups on LFD and HFD; **(A)** SCD-1 (*n* = 5) and **(B)** ATGL (*n* = 4). Results are presented as mean ± SEM. Two-way ANOVA with Fisher's LSD *post hoc* correction was applied and *p* < 0.05 was considered as statistically significant. *p < 0.05, **p < 0.01.

Dox-H mice showed a significant increase in the protein expression of SCD-1 and ATGL compared to vehicle-H (SCD-1, *p* < 0.05 and ATGL, *p* < 0.01) and Dox-L mice (SCD-1, *p* < 0.05 and ATGL, *p* < 0.01), in tumor tissue (Figures 8A,B). Pearson's correlation analysis showed a significantly strong negative correlation between mammary adipose tissue HSL protein expression and plasma resistin concentration in the Dox-H group (*r* = −0.73, *p* < 0.05; Supplementary Figure 2D).

### Mammary Adipose- and Tumor Tissue Fatty Acid Composition

Fatty acids for both mammary adipose (total lipid) and tumor tissue (TPL) are summarized in Supplementary Tables 3, 4, and a select few FAs are presented in graphs. The predominant FA classes in mammary adipose tissue were monounsaturated FAs (MUFAs) ranging from 43 to 51%, followed by SFAs (27–30%) and polyunsaturated FAs (PUFAs; 21–27%) in the treatment groups (Supplementary Figure 3). In the tumor tissue TPL fraction the predominant FA classes were SFAs ranging from 40 to 43%, followed by PUFAs (32–38%) and MUFAs (19–28%) in all the experimental groups (Supplementary Figure 4).

### Diet-Induced Obesity and Doxorubicin Differentially Altered Saturated Fatty Acids in the Tumor Microenvironment

**Figure 9:**
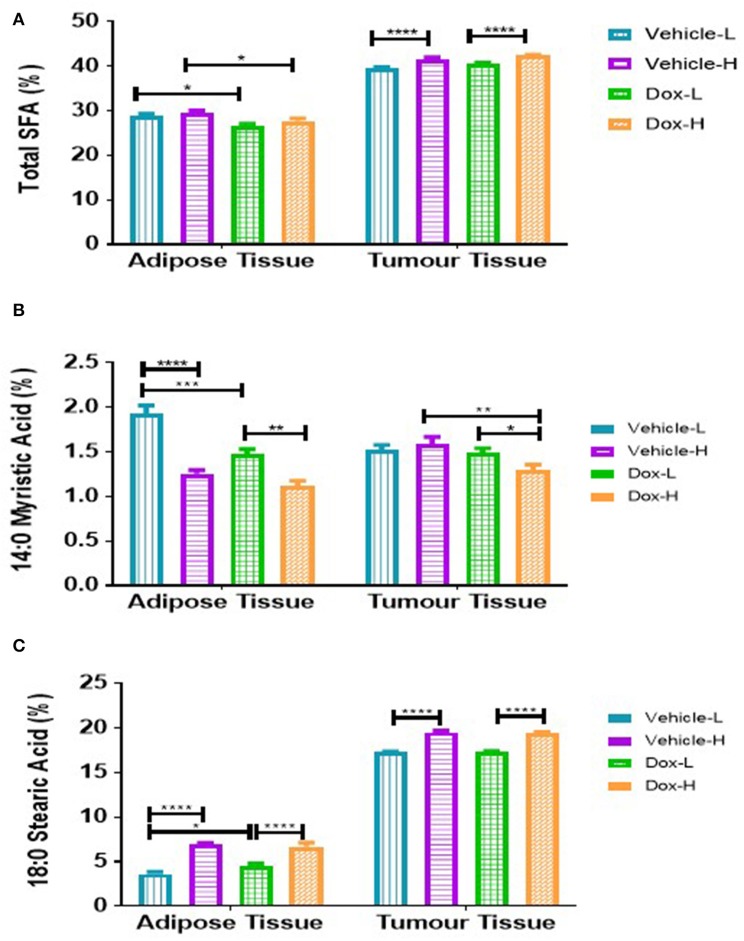
Saturated fatty acid composition; **(A)** Total SFA, **(B)** Myristic Acid, and **(C)** Stearic Acid of mammary adipose- and tumor tissue of mice fed a LFD or HFD with either vehicle control or doxorubicin treatment. Results are presented as mean ± SEM (*n* = 5). Two-way ANOVA with Fisher's LSD *post hoc* correction was applied and *p* < 0.05 was considered as statistically significant. *p < 0.05, **p < 0.01, ***p < 0.001 and ****p < 0.0001.

Total SFAs (Σ SFAs) present in the tumor phospholipid fraction was significantly higher in vehicle-H compared to vehicle-L mice (*p* < 0.0001) and higher in Dox-H compared to Dox-L mice (*p* < 0.0001) (Figure 9A). In mammary adipose tissue, myristic acid (MA, C14:0) was significantly lower in the vehicle-H mice compared to vehicle-L (*p* < 0.0001) and lower in vehicle-L compared to Dox-L mice (*p* < 0.001), respectively (Figure 9B). Myristic acid was also significantly lower in the Dox-H mice compared to Dox-L mice in tumor tissue (*p* < 0.05; Figure 9B). Stearic acid (SA, C18:0) was significantly higher in vehicle-H mice compared to vehicle-L (*p* < 0.0001) in mammary adipose tissue. In addition, SA was also found to be significantly higher in the Dox-H mice compared to Dox-L mice (*p* < 0.0001; Figure 9C). The tumor tissue SA percentage was also higher in vehicle-H compared to vehicle-L mice (*p* < 0.0001) and higher in Dox-H compared to Dox-L mice (*p* < 0.0001), respectively (Figure 9C).

### Diet-Induced Obesity and Doxorubicin Suppressed Monounsaturated Fatty Acids in the Tumor and in Surrounding Mammary Fat

**Figure 10:**
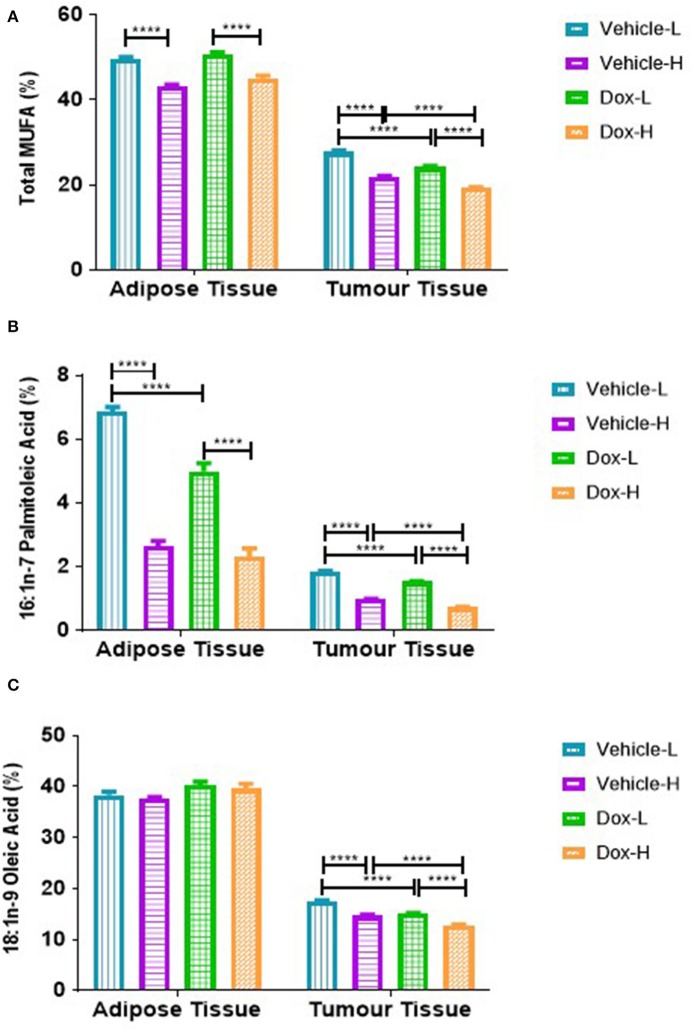
Monounsaturated fatty acid composition; **(A)** Total MUFAs, **(B)** Palmitoleic Acid and **(C)** Oleic acid of mammary adipose- and tumor tissue of mice fed a LFD or HFD diet with either vehicle control or doxorubicin treatment. Results are presented as mean ± SEM (*n* = 5). Two-way ANOVA with Fisher's LSD *post hoc* correction was applied and *p* < 0.05 was considered as statistically significant. ****p < 0.0001.

A similar and significant trend was observed for various MUFAs in both mammary adipose tissue and tumor phospholipid FAs. The total MUFAs (Σ MUFAs) and palmitoleic acid (PTA, C16:1n-7) were significantly lower in vehicle-H compared to vehicle-L mice and significantly lower in Dox-H compared to Dox-L mice, respectively (all *p* < 0.0001, Figures 10A,B). In tumor tissue, oleic acid (OA, C18:1n-9) was significantly lower in vehicle-H vs. vehicle-L and Dox-H vs. Dox-L mice, respectively (*p* < 0.0001, Figure 10C).

### Diet-Induced Obesity and Doxorubicin Selectively Increased Polyunsaturated Fatty Acids in the Tumor Microenvironment

**Figure 11:**
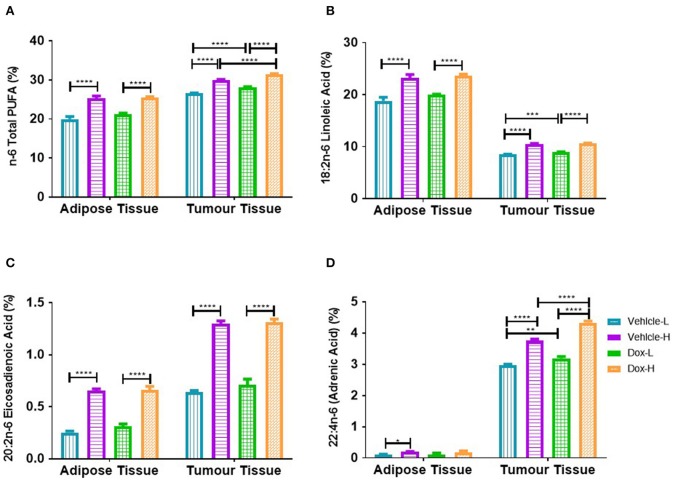
Polyunsaturated fatty acid composition; **(A)** Total n-6 PUFAs, **(B)** Linoleic Acid, **(C)** Eicosadienoic Acid, and **(D)** Adrenic Acid of mammary adipose-and-tumor tissue of mice fed a LFD or HFD with either vehicle control or doxorubicin treatment. Results are presented as mean ± SEM (*n* = 5). Two-way ANOVA with Fisher's LSD *post hoc* correction was applied and *p* < 0.05 was considered as statistically significant. *p < 0.05, **p < 0.01, ***p < 0.001 and ****p < 0.0001.

The total n-6 PUFAs (Σ n-6 PUFAs), linoleic acid (LA, C18:2n-6) and eicosadienoic acid (EDA, C20:2n-6), were significantly higher in the mammary adipose tissue of vehicle-H compared to vehicle-L mice (Σ PUFAs, *p* < 0.0001, LA, *p* < 0.0001, EDA, *p* < 0.0001), and higher in Dox-H than Dox-L mice, respectively (Σ n-6 PUFAs, *p* < 0.0001, LA, *p* < 0.0001, EDA, *p* < 0.0001; Figure 11). Similar results were observed in the tumor tissue total phospholipid FA fraction i.e., higher Σ n-6 PUFAs, LA, EDA and adrenic acid (ADA, C22:4n-6) levels in vehicle-H compared to vehicle-L mice (Σ n-6 PUFAs, *p* < 0.0001, LA, *p* < 0.0001, EDA, *p* < 0.0001, and ADA, *p* < 0.0001) as well as higher percentages of these FAs in Dox-H compared to Dox-L mice (Figure 11).

### Haematoxylin and Eosin Stained Tumors

**Figure 12:**
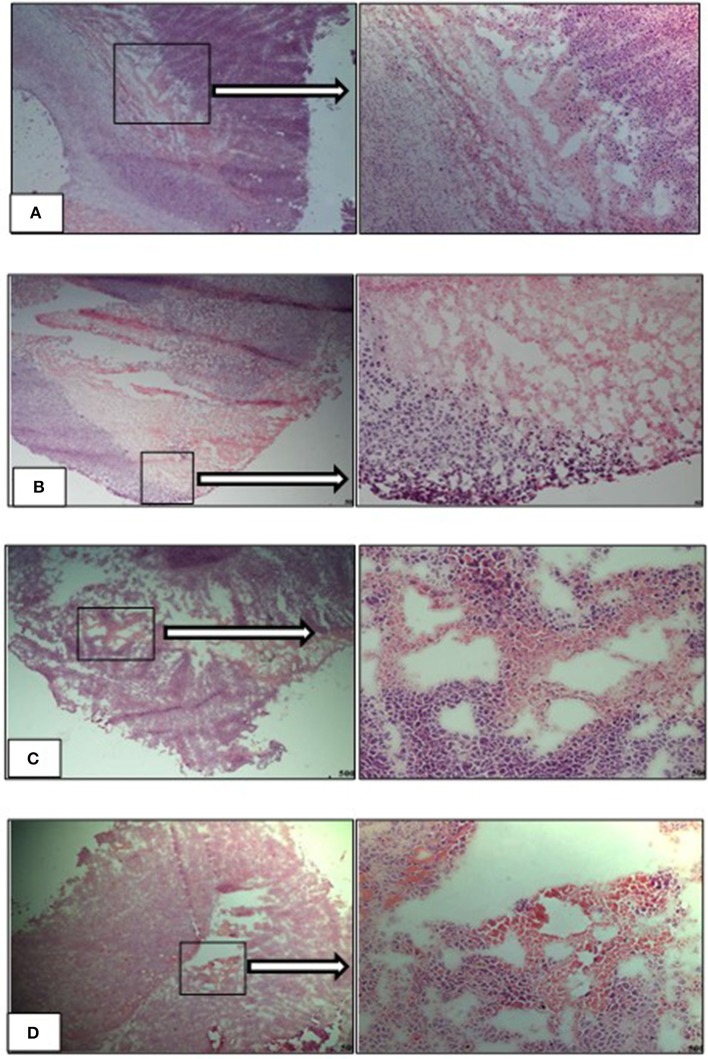
Representative images of H&E stained tumor sections of the experimental treatment groups **(A)** vehicle-LFD, **(B)** vehicle-HFD, **(C)** Dox-L, **(D)** Dox-H, Magnification = 4 and 20 x. Scale = 500 μm.

Necrotic regions were detected in the tumor sections from the vehicle-L mice. Necrosis was identified by cells with pale pink cytoplasm and areas of karyorrhectic debris (Figure 12A). Necrosis was also detected in the tumors from the HFD vehicle treated mice. Viable tumor cells demonstrated hyperchromatic nuclei with coarse chromatin (Figure 12B). Tumors from both the LFD and HFD vehicle mice demonstrated hyper- and hypocellular regions and central areas of necrosis. Tumor sections from the Dox-L mice resembled those of the vehicle-L treated mice. In addition, multinucleated tumor cells were also noted (Figure 12C). Sections from Dox-H mice had a similar appearance (Figure 12D).

## Discussion

### Diet-Induced Obesity Significantly Decreased Doxorubicin Treatment Efficacy in Breast Tumors

Similar to previous findings (33–35) we successfully establish weight gain in our animal model. Body weight of animals in the HFD group was significantly higher than those in the LFD group, before (Figure 2A) and after tumor induction irrespective of treatment (Figure 3A), which was corroborated by mammary adipose tissue weight. As expected, the Dox-H mice had significantly lower food consumption compared to vehicle-H mice (Figure 3B), which can be as a result of a well-known side-effect of doxorubicin treatment (36), however while the loss of appetite was visible, this had no effect on body weight. Furthermore, we reported a significantly higher volume (Figure 5) and weight (Figure 4B) of tumors in the vehicle-H mice and in the Dox-H mice. This is in agreement with previous studies reporting that DIO promoted tumor growth. For example, Lautenbach et al. (37), observed that female obese Sprague Dawley rats (HFD, 60% energy from fat for 8 weeks) were more susceptible to tumor induction by dimethylbenzathracene and also showed increased tumor growth compared to controls. This was corroborated by Khalid et al. (38), who found that a HFD (45% energy from fat) significantly increased body weight and fat mass compared to mice on a LFD (10% energy from fat) in a MMTV-HER2/Neu transgenic breast cancer model, and that obesity promoted tumor growth, reflected by an increase in tumor size. Additionally, Cowen et al. (39) reported a higher body weight in female MMTV-PyMT mice on a HFD (35.7% energy from fat) compared to mice on a LFD (10% energy from fat), even after adjusting for tumor weight and tumor volume. Others reported that DIO promotes tumor growth, progression, and metastasis in animal models (40, 41), specifically in breast cancer (16, 17, 33). In addition, poor treatment outcomes are also reported in overweight and obese breast cancer patients evident by larger tumor sizes and poor clinical outcomes (7, 18, 42, 43) especially those treated with doxorubicin (42, 44). It was also reported that DIO decreased the efficacy of breast cancer treatment protocols in pre-clinical animal models (33, 45).

### Inflammatory Markers: Diet-Induced Obesity Induces Systemic and Mammary Fat Inflammation

Leptin was significantly increased in both the vehicle-H and Dox-H groups compared to the respective control LFD groups (Figure 6C). Leptin concentrations also correlated positively with mammary adipose tissue weight (Supplementary Figure 1). These results indirectly implicate mammary adipose tissue, specifically adipocytes in the tumor microenvironment as a source of leptin secretion in obese mice, which also showed greater mammary adipose weight (Figure 4A). Since E0771 breast cancer cells have been shown not to produce leptin, even when co-cultured with adipocytes, it therefore does not significantly contribute to increased leptin levels (34). The increased mammary adipose tissue weight as a result of the HFD could possibly be one of the primary sources of leptin in our study. We also reported that the resistin concentrations were significantly increased in Dox-H compared to Dox-L mice (Figure 6D).

Obesity is well-known to increase various pro-inflammatory adipokine concentrations in serum and plasma as well as adipose tissue (34, 46), whereas mRNA expression levels showed that adipocytes co-cultivated with breast cancer cells also had significantly higher IL-6, IL-1β, and TNF-α levels (7). It has previously been shown that these elevated circulating cytokines (i.e., IL-6 and IL-8) exerted effects at distant sites (47, 48), this favors the progression of breast cancer by upregulating the secretion of pro-inflammatory adipokines as well as exacerbates immune cell infiltration, which in turn promoted cancer progression through cellular proliferation, angiogenesis and the inhibition of apoptosis (38, 39, 49). Evidence also supports the role of obesity-induced inflammation (IL-6, TNF-α, and leptin) in Tamoxifen® and anti-VEGF acquired breast cancer drug resistance (33, 50).

Leptin and resistin are well-known adipokines linked to breast cancer (51). Both are secreted primarily by adipose tissue, increase with higher degrees of adiposity, and has been implicated for their role in obesity, inflammation, and breast tumorigenesis (51–53). Breast cancer patients are characterized by high serum leptin concentrations as well as increased leptin receptor expression especially in higher pathological grade tumor tissues and patients who develop resistance to anti-cancer treatments (54, 55). Both leptin and resistin exacerbates an inflammatory microenvironment by favoring the secretion of other pro-inflammatory adipokines. Additionally, leptin favors breast cancer progression by inducing cellular proliferation by binding to its receptor followed by downstream signaling through NFκB, STAT3, ERK1/ERK2, and phosphoinositide-3-kinase (PI3K) pathways (56, 57). Both elevated leptin and resistin concentrations was associated with the promotion of cancer stem cell survival and the promotion of invasion and migration via epithelial to mesenchymal transition in breast cancer cells (55, 56, 58), which contributes to the development of treatment resistance.

High concentrations of leptin and resistin favor cancer cell proliferation and have recently been reported to be casual factors in acquired breast cancer treatment resistance (59). A well-known mechanism of developing breast cancer treatment resistance includes the evasion of apoptotic pathways (60, 61). Adipocytes attenuated Doxorubicin-induced apoptosis in cancer cells by increasing the protein expression of anti-apoptotic marker blc-2 as well as increasing the synthesis of resistin (59, 61). Resistin has also been identified as a causal factor for acquiring resistance to doxorubicin treatment in both MCF-7 and MDA-MB-231 breast cancer cells through the induction of autophagy (59).

Furthermore, we also determined the protein expression levels of NFκB, an important transcription factor regulating inflammation, in mammary adipose tissue to confirm local inflammation, and observed significantly higher levels of NFκB-p65 protein expression in Dox-H compared to both the Vehicle-H and Dox-L mice (Figure 7A). Mammary adipose tissue in the tumor microenvironment displays persistent inflammation and harbors crown like structures, which are well-known inflammatory foci (62). We therefore propose that mammary adipose tissue displays local inflammation (as a result of DIO) similar to what is observed in visceral adipose tissue of obese individuals (52, 63) and as a result may play a significant role in obesity-induced breast cancer treatment resistance. It is speculated that treatment resistance may be the result of inflammation found in the mammary adipose tissue as a result of the HFD and doxorubicin treatment. This is confirmed by the fact that doxorubicin treatment induces inflammation in metabolic tissues (64).

We, therefore conclude that inflammation as a result of adipokine dysfunction was observed in obese vehicle-treated mice and to a greater extent in doxorubicin treated mice. We propose that obesity drives both systemic and local inflammation in mammary adipose tissue and thereby induce downstream signaling pathways regulating cell growth, inhibition of apoptosis, and invasion, to ultimately contribute to the development of breast cancer treatment resistance. Therefore, it is plausible that DIO plays a key role as a causal factor in the underlying pathophysiology linked to the decreased efficacy of Doxorubicin treatment, involving systemic and local mammary fat inflammation as underlying molecular mechanisms.

### Diet-Induced Obesity Distinctly Alters Lipid Metabolism in the Tumor Microenvironment Leading to Changes in Fatty Acid Composition in Mammary Adipose- and Tumor Tissue

We found that tumor tissue Σ SFA was increased in both vehicle-H and Dox-H (HFD) mice compared to LFD mice (Figure 9A). Stearic acid (SA) was also found to be increased in mammary adipose tissue and tumor tissue of Dox-H mice compared to Dox-L mice (Figure 9C). Furthermore, we observed decreased percentages of various MUFAs (PTA, OA, and VA) in both mammary adipose and tumor tissue of mice on the HFD, and even more profound decreases in doxorubicin-treated mice (Figure 10).

Clinical and experimental animal model evidence on tumor and adjacent adipose tissue induced FA composition alterations within the tumor microenvironment are lacking specifically under obesogenic conditions. Our results are in agreement with Maillard et al. (65), who showed that the most abundant FAs present in breast cancer tumors were OA, PA, SA, as well as LA, compared to controls. de Bree et al. (66), reported that breast cancer cases showed significantly higher Σ MUFA content in tumor tissue as well as lower Σ PUFAs and n-6 PUFAs content in breast adipose tissue, when compared to benign cases. Mohammadzadeh et al. (67), confirmed increased OA, arachidonic acid (AA) and MUFA:SFA ratio in breast tumors, compared to adjacent tissue.

Due to the abundance and close proximity of mammary adipose tissue (source of FAs) to breast cancer tumors, breast tumors rely on lipid metabolism to favor survival by increasing the expression of various proteins regulating lipid metabolism (68, 69). This is evident by an upregulation of various enzymes catalyzing *de novo* FA synthesis in breast cancer cells i.e., Acetyl-CoA carboxylase (ACC), FAS and SCD-1 (13, 70, 71). Supporting evidence includes increased exogenous lipid utilization, where breast cancer cells induce adipocytes to release FFA via activation of lipolysis (increased expression of ATGL and HSL) and inhibition of adipogenesis (through decreased expression of peroxisome proliferator-activated receptor-γ) (6, 8). Adipocyte-derived FFAs favor the proliferative nature of breast tumor cells in the tumor microenvironment (8), by serving as available metabolic substrates for energy production via β-oxidation or storage in the form of lipid droplets for later utilization. In contrast, microscopically, regions with varying cellularity were observed in tumor sections of both vehicle treated groups irrespective of diet (Figures 12A,B). Hyper-cellular regions also showed darker staining on low-power magnification, indicative of increased cell proliferation. Sections of the Dox-L mice showed mostly hyper-cellular regions (Figure 12C), whereas the tumor sections of the Dox-H mice were less cellular (Figure 12D). This may indicate a decrease in cellular proliferation in response to Doxorubicin treatment among the HFD fed mice. Therefore, it may also be plausible that adipocyte-derived FFAs can induce survival via other mechanisms of action other that proliferation. A recent report showed that lipid accumulation (adipocyte derived FFA) leads to uncoupled FA oxidation, which favored invasion due to epithelial-mesenchymal-transition, but not proliferation (72).

Additionally, adipocyte-derived FFAs can also be incorporated into phospholipids and esterified with cholesterol to produce cholesteryl esters in cell membranes (73, 74) to induce lipid-saturated membranes. This is further supported by the increased amount and size of lipid droplets found in breast cancer tumors, specifically more aggressive phenotypes (6, 73). Therefore, it may also be plausible that the increased tumor volume in the HFD fed groups maybe be as a result of membrane lipid saturation and lipid droplet deposition within tumor tissue. In fact, tumors enriched with lipid droplets (TAGs and sterol esters) were found to be more resistant toward chemotherapeutic agents (75).

Fatty acids are essential components of cell membrane organization (phosphoglycerides) and fluidity (degree of carbon chain unsaturation), and it is known that the type of FA (i.e., increased saturated FAs characteristic of obesity) derived from the diet, affects phospholipid FA composition (densely-packed membranes) and physical-chemical properties (decreased transmembrane permeability) in cancer cells. This metabolic behavior protects breast cancer cells from oxidative damage induced by chemotherapeutic drugs by decreasing lipid peroxidation, ultimately leading to acquired treatment resistance (11, 76).

The decreased SFA and MUFA profiles observed in mammary adipose tissue could possibly be as a result of alterations in the expression of enzymes regulating lipogenesis, since PA can be elongated into SA, as well as desaturated (catalyzed by SCD-1) to produce PTA (77). We found a decrease in FAS (Figure 7B) and SCD-1 protein expression (Figure 7C) in HFD mice (both vehicle- and doxorubicin-treated) within mammary adipose tissue, which translate to a decrease in lipogenic activity in mammary adipose tissue of the HFD (obese) animals. This was further supported by decreased estimated activity of SCD1-16 and SCD1-18 (desaturation indexes) observed in HFD compared to LFD animals in mammary adipose tissue FA composition, specifically in the doxorubicin-treated mice (Supplementary Table 3). Our findings can be explained by the high dietary carbohydrate content of the LFD i.e., 70% energy from carbohydrates, which might partially explain why SCD-1 and FAS expression was higher in the LFD mice, irrespective of treatments, as dietary carbohydrates are substrates for *de novo* FA synthesis. Our results are in agreement with Liu et al. (78), who showed that rats fed a HFD (60% energy from fat) compared to a control diet (10% energy from fat), showed decreased SCD-1 estimated activity derived from FA composition in adipose tissue TAG and serum FFA fractions. Additionally, it may also be possible that a HFD suppresses SCD-1 expression to prevent adipose tissue storage of FAs in order to promote β-oxidation. This could have implications for tumor-cell survival since an increase in β-oxidation is linked to increased energy production which breast cancer cells utilize for survival, and/or to evade the toxic effects of cancer treatments. This provides a plausible explanation for the HFD-induced decreased lipogenic/lipolytic activity in mammary adipose tissue to increase the FFA “pool” by preventing fat storage (TAGs), which is also exacerbated by doxorubicin treatment—all of which may contribute to the attenuation of breast cancer treatment efficacy. We propose that the excess lipid “availability” in mammary adipose tissue of obese patients could explain the resistance to treatment protocols found in breast cancer patients, especially since dysfunctional adipose tissue (obesity) is implicated in breast cancer progression (79) and because “obese” adipocytes provide higher concentrations of FFAs to breast cancer cells to sustain survival and migration (8).

However, the increased SCD-1 expression observed in Dox-H compared to Dox-L mice in tumor tissue (Figure 8A) does not account for the decreased MUFAs found in the tumor tissue of Dox-H mice (Figure 10). Firstly, the decreased MUFA profile may be as a result of increased lipolysis of lipid droplets within the tumor itself, as evident by the increased expression of ATGL in the tumor tissue of the HFD mice (Figure 8B). Additionally, it could also be the result of breast tumor cells utilizing these MUFAs to decrease treatment efficacy, by increasing the release of MUFAs from the cell membrane. This is supported, by the decreased MUFAs observed within the tumor tissue FFA fraction, including PTA, VA, gondoic acid (GA), and nervonic acid (NA), in the Dox-H compared to Dox-L groups (Supplementary Table 5). Lastly, the decreased MUFAs found in tumor tissue TPL and FFA fractions can also be explained by the preferential release and low re-uptake of MUFAs in specific tissues, as well as the selective preference of SFAs compared to MUFAs, or the selective decrease of MUFAs-containing phosphatidylethanolamines and phosphatidylcholine lipids present in tumors under obesogenic conditions. Taken together, the HFD (obesity) induced both *de novo* FA synthesis and lipolysis in the tumor, which was exacerbated by the doxorubicin treatment itself and might therefore confer to the attenuation of breast cancer treatment efficacy under obesogenic conditions.

Furthermore, a dysregulation of cytokines (i.e., increased IL-6, TNF-α, and IL-1β) and adipokines, such as increased leptin and decreased adiponectin (80–83) has been shown to induces transcriptional changes. For example leptin has been shown to inhibit lipogenesis by altering the expression of transcription factors involved in lipid metabolism (7, 45). The outcome is altered adipocyte endocrine functionality which can favor tumor cells to produce more adipokines (83).

We observed increased leptin and resistin levels in Dox-H compared to Dox-L mice (Figures 6C,D). Previously, elevated TNF-α levels have been shown to inhibit adipocyte lipolysis (84) and high leptin levels have been shown to decrease adipose tissue SCD-1 expression (85). We believe that obesity-induced inflammation (increased resistin levels) may lead to lipolysis inhibition (decreased HSL in Dox-H vs. Dox-L mice) in mammary adipose tissue (Figure 7D). In agreement, we found a significant negative correlation between resistin concentration and HSL protein expression in mammary adipose tissue in the Dox-H mice (Supplementary Figure 2), which is supported by previous studies showing that high SFA levels induce the secretion of pro-inflammatory mediators via the NFκB signaling pathway, via the TLR-4 on macrophages (86, 87), which might in turn inhibit lipolysis (decreased HSL). This is also in agreement with our results, since we found higher levels of NFκB protein expression in mammary adipose tissue of Dox-H compared Dox-L mice (Figure 7); all of which further promotes inflammation in the mammary adipose tissue environment, favoring breast cancer cell survival and thereby decreasing treatment efficacy in a paracrine manner.

Furthermore, both mammary adipose tissue and tumor tissue showed significant increases in various n-6 PUFAs (LA, EDA, and ADA) in vehicle-H and Dox-H mice compared to LFD mice (Figure 11). Linoleic acid and ALA are essential FAs derived from the diet (88). These FAs are desaturated (FA desaturases) and elongated (Elovl2 and Elovl5) to form their respective long-chain polyunsaturated products such as AA and eicosapentaenoic acid (EPA) (88). Both the low-fat and high-fat experimental diets in our study contained soybean oil, which is rich in both LA and ALA. Therefore, the increase in PUFAs found in both the mammary adipose tissue and the tumor tissue of the HFD mice may be reflective of the higher total fat content (and therefore PUFA content) of the HFD (60% energy from fat).

The proportions of FAs within the two respective diets differed significantly. Linoleic acid and AA accounted for the elevation of n-6 PUFAs in both mammary and tumor tissue of HFD-fed mice, suggesting an increased inflammatory profile, specifically in the obese doxorubicin-treated mice. The pro-inflammatory effects of n-6-PUFA is as a result of lipid-derived bioactive eicosanoid mediators, such as prostaglandins and leukotrienes (14, 89). These bioactive lipids are implicated in breast cancer progression by favoring angiogenesis, cellular proliferation and survival, cell migration, metastasis, as well as exacerbating inflammation (15, 90), in the breast tumor microenvironment possibly promoting acquired cancer resistance to anti-cancer treatment agents.

More importantly, doxorubicin treatment causes adipose tissue and/or adipocyte dysfunction, by altering lipogenesis (decreased FAS) and lipolysis (increased HSL) (20–22, 64, 91), which participate toward the disruption of adipose tissue homeostasis. The consequence here is an increase in FFA release that disrupts lipid storage (22). Doxorubicin-induced FFA release may further exacerbate the bioavailability of FFA, which cancer cells can utilize favorably for its survival and proliferation demands, and thereby indirectly promote breast cancer treatment resistance. Recently, Ebadi et al. (92), showed that chemotherapy treatment (5-fluorouracil and Irinotecan) in a colorectal cancer model diminished peri-uterine adipose tissues' function to store lipids by significantly downregulating the expression of ACC, FAS, and HSL, as well as markers of β-oxidation (i.e., CPT-2), compared to treatment-naïve rats. Additionally, they also showed that SFAs (PA) and MUFAs (PTA) were significantly decreased in chemotherapy-treated groups. However, the authors explained that it is still unknown whether the suppression of adipose tissue lipid storage capacity induced by chemotherapy is a result of decreased HSL expression, or due to mitochondrial dysfunction induced by the chemotherapy (92). Mehdizadeh et al. (93) showed that doxorubicin and 5-fluorouracil have the ability to induce cancer cell invasion and metastasis by increasing lipid accumulation and membrane fluidity, by altering lipid metabolism. For example, doxorubicin and 5-fluorouracil treatment significantly increased the number of lipid droplets within HepG2 cancer cells. They also reported a significant increase in SFAs (PA) and PUFAs and a significant decrease in MUFAs (OA and PTA) following chemotherapy treatments in the phospholipid fractions of the membranes of cancer cells (93).

To summarize, evidence on FA profiles within the tumor microenvironment has not yet been explored in an obese breast cancer animal model to specifically illustrate its role in breast cancer treatment efficacy. We provide evidence that diet-induced obesity altered the FA profile of both the tumor tissue and its adjacent surrounding mammary adipose tissue. The expression of lipid metabolism enzymes in this study were also differentially altered by diet-induced obesity and it is very likely that the altered FA composition observed in both mammary adipose tissue and tumor tissue are as a result of alterations in lipogenesis and/or lipolysis, which may be a causal factor in decreasing the efficacy of doxorubicin a well-known breast cancer treatment agent. We acknowledge that the fat content used in the HFD (60% energy from fat) of the *in vivo* model is high in comparison to human consumption. However, Ervin et al. (94) suggested that although the total fat content is high, the proportion of specific FA classes (SFAs, MUFAs, and PUFAs) consumed in humans is similar to the HFD we used (94).

## Conclusion

**Figure 13:**
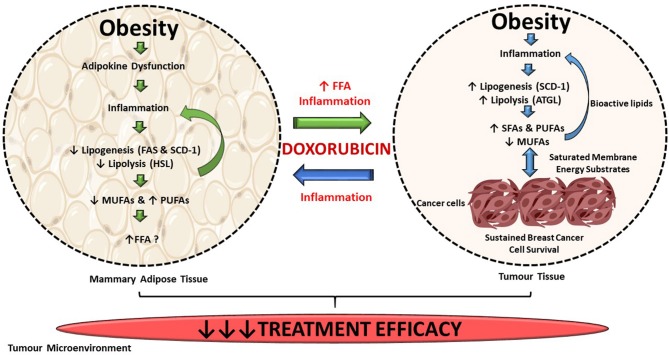
Summary of findings. DIO selectively supresses *de novo* FA synthesis and lipolysis in mammary adipose tissue, but increased lipogenesis and lipolysis in tumor tissue. Exogenous dietary lipids can alter the energy metabolism of triple-negative breast cancer tumors in this current *in vivo* model. Alterations in FAs composition in both mammary adipose and tumor tissue could be a mechanism by which FAs composition can be altered in response to DIO within the tumor microenvironment and thereby contributing to the decreased efficacy of breast cancer treatment agent within our current model.

Diet-induced obesity significantly decreased the treatment efficacy of doxorubicin on triple-negative breast cancer tumors. Suppression of both *de novo* FA synthesis and lipolysis in mammary adipose tissue lead to the inhibition of FA storage (decreased MUFAs and increased PUFAs), exacerbating local inflammation in mammary adipose tissue which can enhance breast cancer cell survival in a paracrine manner, as illustrated in Figure 13. *De novo* FA synthesis and lipolysis were increased in breast tumor tissue. The incorporation of dietary FAs into phospholipid membranes of breast tumor cells suggests that exogenous dietary lipids can alter the energy metabolism of E0771 breast cancer cells. These selective alterations in lipid metabolism markers and FA composition in both mammary adipose and tumor tissue could be a novel mechanism by which FA composition can be altered in response to DIO within the tumor microenvironment and thereby contributing to the development of breast cancer treatment resistance. When doxorubicin is administered as a treatment in an obesogenic context, the treatment efficacy of this breast cancer treatment agent is decreased by conferring to a more lipid saturated cell membrane, known to protect cancer cells from the cytotoxic effects of chemotherapeutic agents.

## Data Availability Statement

All datasets generated for this study are included in the article/Supplementary Material.

## Ethics Statement

The animal study was reviewed and approved by Animal research committee of Stellenbosch University (SU-ACUM13-00015).

## Author Contributions

IM performed and completed all analysis and wrote the first draft of this manuscript. ZE assisted with analysis. LJ completed and assisted with the histological analysis and interpretation of data. TN, PJ, and A-ME contributed to critical revision and intellectual input of the manuscript. All authors read and approved the final manuscript.

### Conflict of Interest

The authors declare that the research was conducted in the absence of any commercial or financial relationships that could be construed as a potential conflict of interest.

## Abbreviations

*Σ* MUFAs: Total Monounsaturated fatty acids
*Σ* n-3 PUFA: Total omega-3 polyunsaturated fatty acids
*Σ* n-6 PUFAs: Total omega-6 polyunsaturated fatty acids
*Σ* PUFAs: Total polyunsaturated fatty acids
*Σ* SFAs: Total saturated fatty acids
AA (C20:4n-6): Arachidonic Acid
ACC: Acetyl-CoA carboxylase
ADA (C22:4n-6): Adrenic Acid
ALA (C18:3n-3): α-Linolenic Acid
ARA (C20:0): Arachidic Acid
ATGL: Adipose triglyceride lipase
CMS: Chloroform:Methanol:Saline
DGLA (C20:3n-6): Dihomo-γ-Linolenic Acid
DHA (C22:6n-3): Docosahexaenoic Acid
DIO: Diet-induced obesity
Dox-H: Tumor doxorubicin-HFD
Dox-L: Tumor doxorubicin-LFD
DPA (C22:5n-6): Docosapentaenoic Acid
EA (C22:1 n-9): Erucic Acid
ECL: Enhanced chemiluminescence
EDA (C20:2n-6): Eicosadienoic Acid
EPA (C20:5n-3): Eicosapentaenoic Acid
FABP4: Fatty acid binding protein 4
FAMEs: Fatty acid methyl esters
GA (C20:1n-9): Gondoic Acid
GLC: Gas-liquid chromatography
HBSS: Hanks Balanced Salt Solution
HFD: High-fat diet
HSL: Hormone-sensitive lipase
IL: Interleukin
LA (C18:2n-6): Linoleic Acid
γ-LA (C18:3n-6): γ-Linolenic Acid
LFD: Low-fat diet
MA (C14:0): Myristic Acid
MCP-1: Macrophage chemoattractant protein-1
MGA (C17:0): Margaric Acid
n-3: Omega-3
n-6: Omega-6
NA (C24:1n-9): Nervonic Acid
NFκB: Nuclear factor kappa B
OA (C18:1n-9): Oleic Acid
PA (C16:0): Palmitic Acid
PAI-1: Plasminogen activator inhibitor-1
PenStrep: Penicillin Streptomycin
PI3K: Phosphoinositide-3-kinase
PTA (C16:1n-7): Palmitoleic Acid
PVDF: Polyvinylidene fluoride
RIPA: Radio-immunoprecipitation assay buffer
SA (C18:0): Stearic Acid
SCD-1: Stearoyl CoA-desaturase-1
SFAs: Saturated fatty acids
SEM: Standard error of the mean
TAG: triacylglycerols
TBS-T: Tris Buffered Saline-Tween 20
TNF-α: Tumor necrosis factor-alpha
TPL: Total phospholipid
VA (C18:1n-7): cis-Vaccenic Acid
VEGF: Vascular endothelial growth factor
Vehicle-H: Tumor vehicle-HFD
Vehicle-L: Tumor vehicle-LFD.
