# Supplementary material for: Decreased Efficacy of Doxorubicin Corresponds With Modifications in Lipid Metabolism Markers and Fatty Acid Profiles in Breast Tumors From Obese vs. Lean Mice
Source: Front Oncol. 2020 Mar 17;10:306. doi: 10.3389/fonc.2020.00306 (PMC7089940; doi:10.3389/fonc.2020.00306)
Supplement: Supplementary file 1 [file Table_1.docx]

## SUPPLEMENTARY

**Supplementary Table 1:** Typical fatty acid composition of LFD (Research diets, D12450J) and HFD (Research Diets, D12492).

| **Fatty acid composition** | **LFD** (D12450J) | **HFD** (D12492) |
| --- | --- | --- |
| Saturated (g) | 10,1 | 81,5 |
| Monounsaturated (g) | 12,8 | 91,5 |
| Polyunsaturated (g) | 20,2 | 81,5 |
|  |  |  |
| Saturated (%) | 23,5 | 32 |
| Monounsaturated (%) | 29,7 | 35,9 |
| Polyunsaturated (%) | 46,8 | 32 |
|  |  |  |
| **Fatty Acid** |  |  |
| [Acetic Acid](https://en.wikipedia.org/wiki/Propionic_acid) | 0 | 0 |
| [Butyric acid](https://en.wikipedia.org/wiki/Butyric_acid) | 0 | 0 |
| [Caproic acid](https://en.wikipedia.org/wiki/Caproic_acid) | 0 | 0 |
| [Caprylic acid](https://en.wikipedia.org/wiki/Caprylic_acid) | 0 | 0 |
| [Capric acid](https://en.wikipedia.org/wiki/Capric_acid) | 0 | 0,1 |
| [Lauric acid](https://en.wikipedia.org/wiki/Lauric_acid) | 0 | 0,2 |
| [Myristic acid](https://en.wikipedia.org/wiki/Myristic_acid) | 0,3 | 2,8 |
| [Pentadecanoic acid](https://en.wikipedia.org/wiki/Pentadecylic_acid) | 0 | 0,2 |
| [Palmitic acid](https://en.wikipedia.org/wiki/Palmitic_acid) | 6,4 | 49,9 |
| Palmitoleic acid | 0,3 | 3,4 |
| [Margaric acid](https://en.wikipedia.org/wiki/Margaric_acid) | 0,1 | 0,9 |
| [Stearic acid](https://en.wikipedia.org/wiki/Stearic_acid) | 3,1 | 26,9 |
| Oleic acid | 12,3 | 86,6 |
| Linoleic acid | 17,8 | 73,1 |
| Linolenic acid | 2,1 | 5,2 |
| [Arachidic acid](https://en.wikipedia.org/wiki/Arachidic_acid) | 0,1 | 0,4 |
| Eicosenoic acid (C20:1) | 0,2 | 1,5 |
| Eicosadienoic acid (C20:2) | 0,2 | 2 |
| Dihomo-γ-Linolenic acid | 0 | 0,3 |
| Arachidonic acid | 0,1 | 0,7 |
| Eicosapentaenoic acid | 0 | 0 |
| Behenic acid | 0,1 | 0 |
| [Docosapentaenoic acid](https://en.wikipedia.org/wiki/Docosapentaenoic_acid) | 0 | 0,2 |
| Total | 43,1 | 254,5 |

As per manufacturer product data sheet (Research diet Inc., New Jersey, USA).

**Supplementary Table 2**: Antibodies used for western blot analysis.

| Primary Antibody | Size | Concentration | Supplier |
| --- | --- | --- | --- |
| Fatty acid synthase (FAS) | 273 kDa | 1:1000 | Cell Signalling (#3180) |
| Stearoyl-CoA desaturase (SCD-1) | 37 kDa | 1:1000 | Cell Signalling (#2794) |
| Adipose tissue triglyceride lipase (ATGL) | 54 kDa | 1:1000 | Cell Signalling (#2439) |
| Hormone sensitive lipase (HSL) | 81-83 kDa | 1:1000 | Cell Signalling (#4107) |
| Fatty acid binding protein 4 (FABP4) | 15 kDa | 1:1000 | Cell Signalling (#3544) |
| Nuclear factor kappa-light-chain-enhancer of activated B cells (NFĸB-p65) | 65 kDa | 1:1000 | Cell Signalling (#8242) |
| Secondary Antibody |  |  |  |
| Anti-rabbit |  | 1:10 000 | Cell Signalling (#7074) |

**Supplementary Table 3:** Mammary Adipose tissue total lipid fatty acid percentage composition of mice from the different experimental groups.

| **Adipose Tissue fatty acid percentage** | | | | | **Significance** | | | |
| --- | --- | --- | --- | --- | --- | --- | --- | --- |
| **Fatty Acid** | **Vehicle-L (%)** | **Vehicle-H (%)** | **Dox-L (%)** | **Dox-H (%)** | **Vehicle-L**  ***vs***  **Vehicle-H** | **Vehicle-L *vs***  **Dox-L** | **Vehicle-H**  ***vs***  **Dox-H** | **Dox-H**  ***vs***  **Dox-L** |
| **SFAs** | | | | | | | | |
| **∑ SFAs** | 29,28 ± 0,43 | 30,03 ± 0,68 | 27,18 ± 0,40 | 28,26 ± 0,62 | NS | * | * | NS |
| **14:0 (Myristic Acid, MA)** | 1,93 ± 0,095 | 1,25± 0,041 | 1,48 ± 0,055 | 1,11 ± 0,062 | **** | *** | NS | ** |
| **16:0 (Palmitic Acid, PA)** | 23,27 ± 0,30 | 21,13 ± 0,45 | 20,6 ± 0,14 | 19,73 ± 0,86 | ** | ** | NS | * |
| **18:0 (Stearic Acid, SA)** | 3,62 ± 0,25 | 6,88 ± 0,22 | 4,48 ± 0,33 | 6,69 ± 0,46 | **** | * | **** | **** |
| **20:0 (Arachidic Acid, ARA)** | 0,13 ± 0,015 | 0,17 ± 0,025 | 0,20 ± 0,022 | 0,14 ± 0,027 | NS | * | NS | NS |
| **MUFAs** | | | | | | | | |
| **∑ MUFAs** | 49,65 ± 0,46 | 43,36 ± 0,18 | 50,64 ± 0,61 | 44,95 ± 0.80 | **** | NS | NS | **** |
| **∑ n-7 MUFAs** | 10,41 ± 0.22 | 4,94 ± 0,23 | 9,102 ± 0,34 | 4,574 ± 0,29 | **** | ** | NS | **** |
| **16:1n-7 (Palmitoleic Acid, PTA)** | 6,86 ± 0,15 | 2,65 ± 0,15 | 4,98 ± 0,27 | 2,32 ± 0,26 | **** | **** | NS | **** |
| **18:1n-7 ( *cis*-Vaccenic Acid, VA)** | 3,54 ± 0,10 | 2,29 ± 0,16 | 4,13 ± 0,11 | 2,26 ± 0,13 | ** | **** | NS | **** |
| **∑ n-9 MUFAs** | 39,24 ± 0,66 | 38,42 ± 0,37 | 41,53 ± 0,70 | 40,37 ± 1,06 | NS | * | NS | NS |
| **18:1n-9 (Oleic Acid, OA)** | 38,29 ± 0,71 | 37,57 ± 0,37 | 40,2 ± 0,72 | 39,55 ± 1,00 | NS | NS | NS | NS |
| **20:1n-9 (Gondoic Acid, GA)** | 0,94 ± 0,04 | 0,80 ± 0,04 | 1,21 ± 0,09 | 0,79 ± 0,08 | NS | ** | NS | *** |
| **PUFAs** | | | | | | | | |
| **∑ PUFAs** | 21,09 ± 0,69 | 26,61 ± 0,68 | 22,18 ± 0,35 | 26,79 ± 0,24 | **** | NS | NS | **** |
| **∑ n-3 PUFAs** | 1,07 ± 0.054 | 1,36 ± 0,039 | 0,89 ± 0,13 | 1,25 ± 0,096 | * | NS | NS | ** |
| **18:3n-3 (α-Linolenic Acid, ALA)** | 0,85 ± 0,041 | 0,76 ± 0,032 | 0,64 ± 0,029 | 0,72 ± 0,065 | NS | ** | NS | NS |
| **22:6n-3 (Docosahexaenoic Acid, DHA)** | 0,19 ± 0,016 | 0,34 ± 0,018 | 0,18 ± 0,042 | 0,28 ± 0,038 | ** | NS | NS | * |
| **∑ n-6 PUFAs** | 20,00 ± 0,66 | 25,25 ± 0,64 | 21,29 ± 0,23 | 25,54 ± 0,19 | **** | NS | NS | **** |
| **18:2n-6 (Linoleic Acid, LA)** | 18,82 ± 0,70 | 23,27 ± 0,61 | 19,99 ± 0,11 | 23,71 ± 0,20 | **** | NS | NS | **** |
| **18:3n-6 (γ-Linolenic Acid, γ-LA )** | 0,17 ± 0,018 | 0,16 ± 0,0055 | 0,17 ± 0,011 | 0,16 ± 0,014 | NS | NS | NS | NS |
| **20:2n-6 (Eicosadienoic Acid, EDA)** | 0,25 ± 0,013 | 0,65 ± 0,019 | 0,31 ± 0,022 | 0,66 ± 0,038 | **** | NS | NS | **** |
| **20:3n-6 (Dihomo-γ-Linolenic Acid, DGLA)** | 0,16 ± 0,010 | 0,24 ± 0,010 | 0,17 ± 0,015 | 0,23 ± 0,013 | *** | NS | NS | ** |
| **20:4n-6 (Arachidonic Acid, AA)** | 0,044 ± 0,25 | 0,58 ± 0,0098 | 0,46 ± 0,043 | 0,56 ± 0,069 | * | NS | NS | * |
| **22:4n-6 (Adrenic Acid, ADA)** | 0,11 ± 0,017 | 0,20 ± 0,0073 | 0,12 ± 0,038 | 0,18 ± 0,045 | * | NS | NS | NS |
| **22:5n-6 (Docosapentaenoic Acid, DPA)** | 0,080 ± 0,0058 | 0,12 ± 0,0037 | 0,090 ± 0,040 | 0,042 ± 0,014 | * | NS | NS | * |
| **Estimated desaturase enzyme activity** | | | | | | | | |
| **SCD1-16** | 0,30 ± 0,010 | 0,13 ± 0,007 | 0,24 ± 0,012 | 0,12 ± 0,009 | **** | ** | NS | **** |
| **SCD1-18** | 10,83 ± 0.90 | 5,49 ± 0,19 | 9,18 ± 0.72 | 6, 00 ± 0.36 | **** | NS | NS | ** |

Results are presented as mean ± SEM (n=5 per group) where Two-way ANOVA with Fishers LSD *post hoc* correction was employed. p<0.05 was considered as statistically significant. *= p<0.05, **= p<0.01, *** = p<0.001 and **** p<0.0001. Abbreviations: Vehicle-L, tumour vehicle-LFD; Vehicle-H, tumour vehicle-HFD; Dox-L, tumour doxorubicin-LFD; Dox-H, tumour doxorubicin-HFD; SCD-1, stearoyl-CoA desaturase-1 (estimated desaturase activity using product-to-precursor FA ratios: SCD1-16 = 16:1n-7:/16:0 ratio; SCD1-18 = 18:1n-9/:18:0 ratio).

**Supplementary Table 4:** Tumour tissue total phospholipid fatty acid percentage composition of mice from the different all experimental groups.

| **Tumour TPL fatty acid percentage** | | | | | **Significance** | | | |
| --- | --- | --- | --- | --- | --- | --- | --- | --- |
| **Fatty Acid** | **Vehicle-L (%)** | **Vehicle-H (%)** | **Dox-L (%)** | **Dox-H (%)** | Vehicle-L  *vs*  Vehicle-H | Vehicle-L  *vs*  Dox-L | Vehicle-H  *vs*  Dox-H | Dox-H  *vs*  Dox-L |
| **SFAs** | | | | | | | | |
| **∑ SFAs** | 39,67 ± 0,17 | 41,57 ± 0,46 | 40,52 ± 0,27 | 42,38 ± 0,17 | *** | NS | NS | *** |
| **14:0 (Myristic Acid, MA)** | 1,53 ± 0,050 | 1,59 ± 0,079 | 1,49 ± 0,053 | 1,30 ± 0,061 | NS | NS | ** | * |
| **16:0 (Palmitic Acid, PA)** | 19,76 ± 0,22 | 19,47 ± 0,30 | 20,65 ± 0,20 | 20,53 ± 0,24 | NS | * | ** | NS |
| **18:0 (Stearic Acid, SA)** | 17,3 ± 0,067 | 19,52 ± 0,19 | 17,28 ± 0,10 | 19,46 ± 0,10 | **** | NS | NS | **** |
| **20:0 (Arachidic Acid, ARA)** | 0,033 ± 0,033 | 0,11 ± 0,077 | 0,097 ± 0,097 | 0,11 ± 0,069 | NS | NS | NS | NS |
| **MUFAs** | | | | | | | | |
| **∑ MUFAs** | 27,76 ± 0,31 | 21,88 ± 0,28 | 24,24 ± 0,17 | 19,28 ± 0,20 | **** | **** | **** | **** |
| **∑ n-7 MUFAs** | 6,49 ± 0,047 | 4,13 ± 0,035 | 5,53 ± 0,084 | 3,45 ± 0,064 | **** | **** | **** | **** |
| **16:1n-7 (Palmitoleic Acid, PTA)** | 1,84 ± 0,027 | 0,98 ± 0,016 | 1,54 ± 0,010 | 0,72 ± 0,015 | **** | **** | **** | **** |
| **18:1n-7 ( *cis*-Vaccenic Acid, VA)** | 4,65 ± 0,026 | 3,15 ± 0,025 | 4,30 ± 0,079 | 2,72 ± 0,050 | **** | *** | **** | **** |
| **∑ n-9 MUFAs** | 21,27 ± 0,28 | 17,75 ± 0,27 | 18,41 ± 0,14 | 15,84 ± 0,16 | **** | **** | **** | **** |
| **18:1n-9 (Oleic Acid, OA)** | 17,47 ± 0,25 | 14,69 ± 0,21 | 15,04 ± 0,10 | 12,85 ± 0,13 | **** | **** | **** | **** |
| **20:1n-9 (Gondoic Acid, GA)** | 1,11 ± 0,025 | 1,07 ± 0,021 | 0,93 ± 0,023 | 0,80 ± 0,027 | NS | **** | **** | ** |
| **22:1 n-9 (Erucic Acid, EA)** | 0,30 ± 0,0066 | 0,27 ± 0,018 | 0,25 ± 0,0072 | 0,24 ± 0,016 | NS | * | NS | NS |
| **24:1n-9 (Nervonic Acid, NA)** | 2,39 ± 0,062 | 1,73 ± 0,093 | 2,18 ± 0,072 | 2,00 ± 0,083 | **** | NS | * | NS |
| **PUFAs** | | | | | | | | |
| **∑ PUFAs** | 32,57 ± 0,25 | 36,55 ± 0,24 | 35,24 ± 0,31 | 38,34 ± 0,12 | **** | **** | *** | **** |
| **∑ n-3 PUFAs** | 6,00 ± 0,14 | 6,64 ± 0,15 | 7,06 ± 0,27 | 6,86 ± 0,10 | * | *** | NS | NS |
| **22:5n-3 (Docosapentaenoic Acid, DPA)** | 1,04 ± 0,0079 | 1,20 ± 0,027 | 1,08 ± 0,019 | 1,32 ± 0,12 | NS | NS | NS | * |
| **22:6n-3 (Docosahexaenoic Acid, DHA)** | 4,95 ± 0,14 | 5,31 ± 0,10 | 5,89 ± 0,33 | 5,5 ± 0,09 | NS | ** | NS | NS |
| **∑ n-6 PUFAs** | 26,57 ± 0,14 | 29,92 ± 0,23 | 28,18 ± 0,10 | 31,48 ± 0,14 | **** | **** | **** | **** |
| **18:2n-6 (Linoleic Acid, LA)** | 8,51 ± 0,05 | 10,5 ± 0,12 | 9,02 ± 0,01 | 10,63 ± 0,09 | **** | *** | NS | **** |
| **20:2n-6 (Eicosadienoic Acid, EDA)** | 0,65 ± 0,011 | 1,30 ± 0,027 | 0,71 ± 0,059 | 1,31 ± 0,034 | **** | NS | NS | **** |
| **20:3n-6 (Dihomo-γ-Linolenic Acid, DGLA)** | 1,73 ± 0,030 | 1,64 ± 0,026 | 1,59 ± 0,017 | 1,47 ± 0,025 | * | ** | *** | ** |
| **20:4n-6 (Arachidonic Acid, AA)** | 12,27 ± 0,12 | 12,31 ± 0,087 | 13,11 ± 0,18 | 13,36 ± 0,092 | NS | **** | **** | NS |
| **22:4n-6 (Adrenic Acid, ADA)** | 2,98 ± 0,025 | 3,76 ± 0,053 | 3,19 ± 0,060 | 4,33 ± 0,057 | **** | ** | **** | **** |
| **22:5n-6 (Docosapentaenoic Acid, DPA)** | 0,43 ± 0,02 | 0,40 ± 0,02 | 0,49 ± 0,01 | 0,38 ± 0,01 | * | NS | NS | *** |
| **Estimated desaturase enzyme activity** | | | | | | | | |
| **SCD1-16** | 0,093 ± 0,0021 | 0,050 ± 0,0010 | 0,074 ± 0,00061 | 0,035 ± 0,0011 | **** | **** | **** | **** |
| **SCD1-18** | 1,01 ± 0,012 | 0,75 ± 0,014 | 0,87 ± 0,0048 | 0,66 ± 0,081 | **** | **** | **** | **** |

Results are presented as mean ± SEM (n=5) where Two-way ANOVA with Fishers LSD post hoc correction was employed, p<0.05 was considered as statistically significant. *= p<0.05, **= p<0.01, *** = p<0.001 and **** p<0.0001. Abbreviations: Vehicle-L, tumour vehicle-LFD; Vehicle-H, tumour vehicle-HFD; Dox-L, tumour doxorubicin-LFD; Dox-H, tumour doxorubicin-HFD; PUFAs, SCD-1, stearoyl-CoA desaturase-1 (estimated desaturase activity using product-to-precursor FA ratios: SCD1-16 = 16:1n-7:/16:0 ratio; SCD1-18 = 18:1n-9/:18:0 ratio).

**Supplementary Table 5:** Tumour free fatty acid percentage composition of mice from the different experimental groups.

| **Tumour FFA percentage** | | | | | **Significance** | | | |
| --- | --- | --- | --- | --- | --- | --- | --- | --- |
| **Fatty Acid** | **Vehicle-L (%)**  **(n=5)** | **Vehicle-H (%)**  **(n=5)** | **Dox-L (%)**  **(n=5)** | **Dox-H (%)**  **(n=5)** | **Vehicle-L**  ***vs***  **Vehicle-H** | **Vehicle-L *vs***  **Dox-L** | **Vehicle-H**  ***vs***  **Dox-H** | **Dox-H**  ***vs***  **Dox-L** |
| **SFAs** | | | | | | | | |
| **∑ Total SFA** | 38,88 ± 1,01 | 44,38 ± 1,36 | 42,02 ± 0,52 | 44,38 ± 1,09 | ** | * | NS | NS |
| **14:0 (Myristic Acid, MA)** | 1,56 ± 0,083 | 1,13 ± 0,21 | 1,23 ± 0,078 | 1,31 ± 0,077 | * | NS | NS | NS |
| **16:0 (Palmitic Acid, PA)** | 21,77 ± 0,50 | 21,33 ± 0,70 | 21,7 ± 0,43 | 22,91 ± 0,49 | NS | NS | * | NS |
| **18:0 (Stearic Acid, SA)** | 15,17 ± 0,36 | 21,81 ± 1,01 | 19,09 ± 0,78 | 20,16 ± 0,91 | **** | ** | NS | NS |
| **MUFAs** | | | | | | | | |
| **∑ MUFAs** | 39,09 ± 0,41 | 30,28 ± 0,80 | 34,07 ± 0,65 | 31,22 ± 0,74 | **** | **** | NS | ** |
| **∑ n-7 MUFAs** | 8,18 ± 0,06 | 3,96 ± 0,23 | 6,59 ± 0,13 | 3,68 ± 0,07 | **** | **** | NS | **** |
| **16:1n-7 (Palmitoleic Acid, PTA)** | 37,9 ± 0,012 | 1,43 ± 0,11 | 2,54 ± 0,14 | 1,45 ± 0,054 | **** | **** | NS | **** |
| **18:1n-7 (*cis*-Vaccenic Acid, VA)** | 4,39 ± 0,061 | 2,53 ± 0,14 | 4,05 ± 0,045 | 2,23 ± 0,044 | **** | ** | * | **** |
| **∑ n-9 MUFAs** | 30,91 ± 0,36 | 26,32 ± 0,69 | 27,48 ± 0,56 | 27,54 ± 0,69 | **** | *** | NS | NS |
| **18:1n-9 (Oleic Acid, OA)** | 28,97 ± 0,34 | 24,54 ± 0,79 | 25,24 ± 0,58 | 26,07 ± 0,66 | *** | *** | NS | NS |
| **20:1n-9 (Gondoic Acid, GA)** | 1,07 ± 0,03 | 1,07 ± 0,04 | 1,11 ± 0,04 | 0,94 ± 0,03 | NS | NS | * | ** |
| **24:1n-9 (Nervonic Acid, NA)** | 0,88 ± 0,025 | 0,89 ± 0,078 | 1,12 ± 0,076 | 0,88 ± 0,020 | NS | ** | NS | * |
| **PUFAs** | | | | | | | | |
| **∑ PUFAs** | 22,03 ± 0,64 | 25,34 ± 0,86 | 23,91 ± 0,45 | 24,41 ± 0,45 | ** | * | NS | NS |
| **∑ n-3 PUFAs** | 2,04 ± 0,18 | 2,38 ± 0,33 | 2,61 ± 0,21 | 1,82 ± 0,035 | NS | NS | NS | * |
| **22:6n-3 (Docosahexaenoic Acid, DHA)** | 1,92 ± 0,06 | 2,22 ± 0,18 | 2,35 ± 0,08 | 1,82 ± 0,04 | NS | * | * | ** |
| **∑ n-6 PUFAs** | 19,99 ± 0,48 | 22,96 ± 0,56 | 21,3 ± 0,31 | 22,59 ± 0,43 | *** | NS | NS | NS |
| **18:2n-6 (Linoleic Acid, LA)** | 10,59 ± 0,24 | 12,07 ± 0,48 | 9,69 ± 0,30 | 12,90 ± 0,24 | ** | * | NS | **** |
| **20:2n-6 (Eicosadienoic Acid, EDA)** | 0,71 ± 0,018 | 1,38 ± 0,055 | 0,85 ± 0,042 | 1,41 ± 0,041 | **** | * | NS | **** |
| **20:3n-6 (Dihomo-γ-Linolenic Acid, DGLA)** | 0,92 ± 0,04 | 0,92 ± 0,06 | 1,04 ± 0,04 | 0,88 ± 0,05 | NS | NS | NS | 0.062 |
| **20:4n-6 (Arachidonic Acid, AA)** | 6,30 ± 0,11 | 6,62 ± 0,42 | 7,62 ± 0,33 | 6,59 ± 0,02 | NS | ** | NS | * |
| **22:4n-6 (Adrenic Acid, ADA)** | 1,61 ± 0,039 | 1,97 ± 0,13 | 2,11 ± 0,083 | 1,98 ± 0,073 | *** | *** | NS | NS |
| **Estimated desaturase enzyme activity** | | | | | | | | |
| **SCD1-16** | 0,17 ± 0,0042 | 0,067 ± 0,0056 | 0,12 ± 0,0047 | 0,063 ± 0,0020 | **** | **** | NS | **** |
| **SCD1-18** | 1,92 ± 0,067 | 1,14 ± 0,077 | 1,34 ± 0,082 | 1,31 ± 0,089 | ***** | **** | NS | NS |

Results are presented as mean ± SEM (n=5 per group) where Two-way ANOVA with Fishers LSD *post hoc* correction was employed. p<0.05 was considered as statistically significant. *= p<0.05, **= p<0.01, *** = p<0.001 and **** p<0.0001. Abbreviations: Vehicle-L, tumour vehicle-LFD; Vehicle-H, tumour vehicle-HFD; Dox-L, tumour doxorubicin-LFD; Dox-H, tumour doxorubicin-LFD; SFAs, saturated fatty acids; MUFAs, monounsaturated fatty acids; PUFAs, polyunsaturated fatty acids; SCD-1, stearoyl-CoA desaturase-1 (estimated desaturase activity using product-to-precursor FA ratios: SCD1-16 = 16:1n-7:/16:0 ratio; SCD1-18 = 18:1n-9/:18:0 ratio).

**Supplementary Figure 1**: Correlations between plasma leptin concentration and mammary adipose tissue weight for vehicle (A&B) and doxorubicin treated groups (C&D) on LFD and HFD diets.

**Supplementary Figure 2:** Correlations between the plasma resistin and mammary adipose tissue hormone sensitive lipase (HSL) protein expression for vehicle (A&B) and doxorubicin (C&D) treated groups on LFD and HFD.


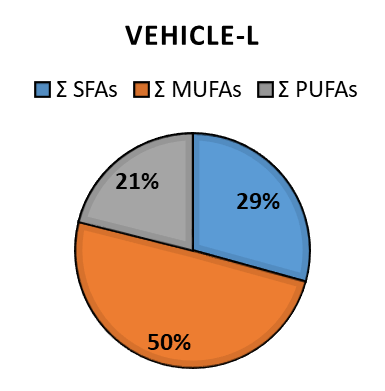

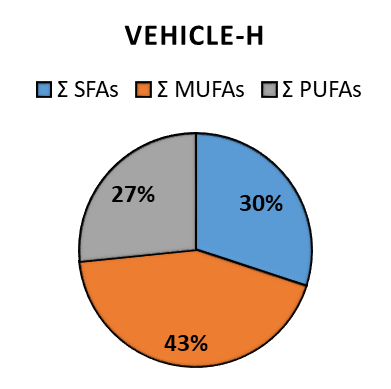


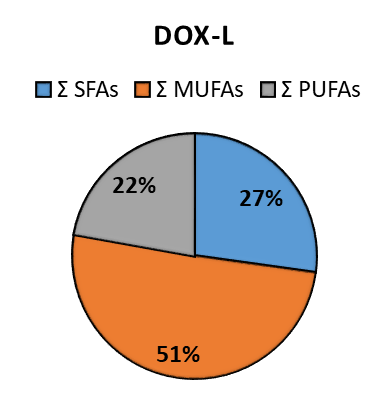

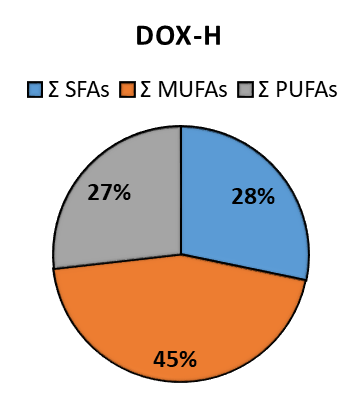


**Supplementary Figure 3:** Summary of fatty acid class percentages in mammary adipose tissue total lipid for all experimental groups.


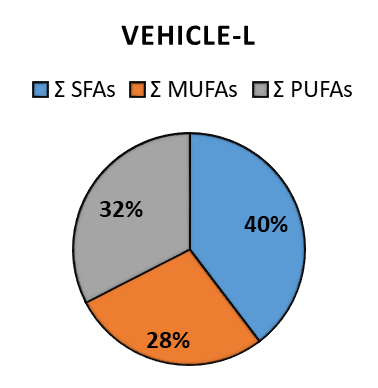

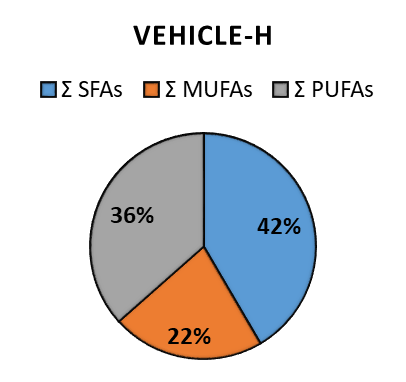


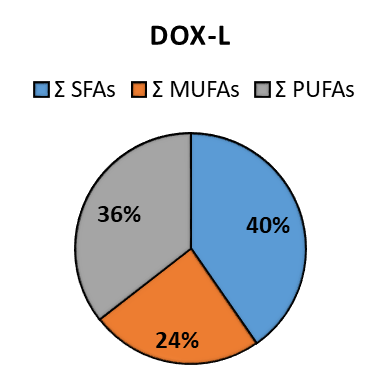

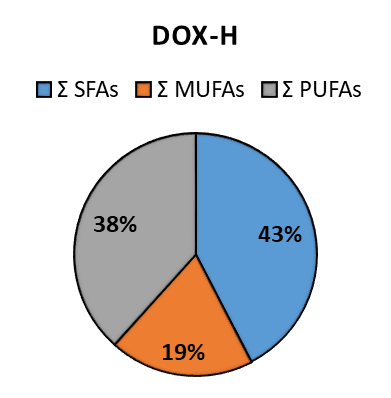


**Supplementary Figure 4**: Summary of fatty acid class percentages in the tumour tissue total phospholipid fraction for all experimental groups.


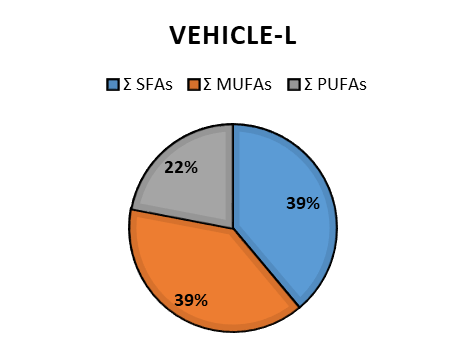

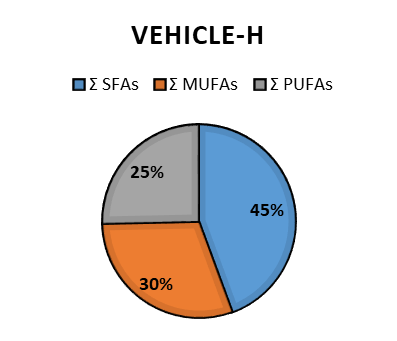

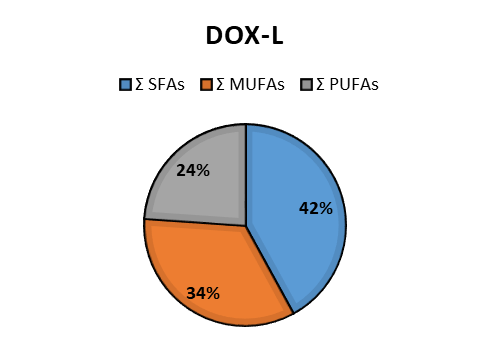

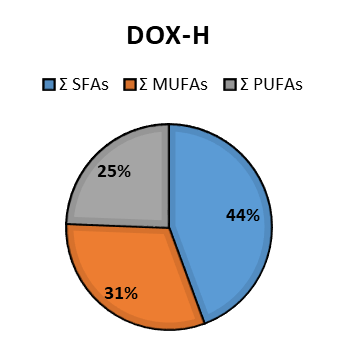


**Supplementary Figure 5**: Summary of tumour tissue free fatty acid class percentages for all experimental groups.
